# Interfacial Redox Engineering of TiO_2_ Nanocomposites Using Green Tea-Derived Ligands and Silver

**DOI:** 10.3390/molecules31173123

**Published:** 2026-09-06

**Authors:** Valentina Nikšić, Dušan Sredojević, Miriama Malček Šimunková, Andrea Pirković, Ana Milivojević, Vlasta Brezová, Vesna Lazić

**Affiliations:** 1 Centre of Excellence for Photoconversion, Vinča Institute of Nuclear Sciences, University of Belgrade, 11000 Belgrade, Serbia; valentina.niksic@vin.bg.ac.rs (V.N.);; 2 Department of Physical Chemistry, Institute of Physical Chemistry and Chemical Physics, Faculty of Chemical and Food Technology, Slovak University of Technology in Bratislava, 812 37 Bratislava, Slovakia; miriama.simunkova@stuba.sk (M.M.Š.); vlasta.brezova@stuba.sk (V.B.); 3 INEP Institute for Application of Nuclear Energy, University of Belgrade, 11000 Belgrade, Serbia; andrea.pirkovic@inep.co.rs; 4 Faculty of Technology and Metallurgy, University of Belgrade, 11000 Belgrade, Serbia

**Keywords:** TiO_2_ surface modification, interfacial charge transfer, green tea-derived surface ligands, redox-active nanocomposites, antimicrobial activity

## Abstract

Titanium dioxide (TiO_2_) is a widely studied semiconductor whose interfacial redox properties strongly influence its photocatalytic and biological performance. In this work, TiO_2_ nanomaterials were surface-functionalized with green tea waste extract (GT) and subsequently impregnated with silver to obtain redox-active nanocomposites with tunable optical and biological properties. HPLC, FTIR, diffuse reflectance spectroscopy (DRS), and density functional theory (DFT) analyses demonstrated the formation of an organic–inorganic interface through adsorption of green tea-derived ligands. DFT calculations revealed complementary interfacial roles of the adsorbed constituents, with epigallocatechin gallate (EGCG) inducing interfacial charge-transfer (ICT) states that enable visible-light absorption, as reflected by the decrease in the apparent optical bandgap from ~3.48 eV for pristine TiO_2_ to ~1.83 eV for TiO_2_/EGCG. ICP-OES analysis further quantified the Ag loading in TiO_2_/GT/Ag at 5.1 wt%. Electron paramagnetic resonance (EPR) experiments demonstrated that GT functionalization shifts the interfacial redox balance toward radical scavenging by suppressing excessive radical generation, whereas silver incorporation partially restores oxidative pathways, particularly under irradiation. These differences in interfacial redox behavior directly translate into distinct biological responses. TiO_2_/GT/Ag showed the strongest antimicrobial activity, with visible-light enhancement observed predominantly against *Staphylococcus aureus*, while TiO_2_/GT/Ag reduced H_2_O_2_-induced oxidative stress in non-malignant cells and promoted intracellular reactive oxygen species generation in cancer cells. These findings demonstrate that engineering the organic–inorganic interface through plant-derived ligands and silver incorporation provides an effective strategy for tuning the interfacial redox properties and light-responsive biological performance of TiO_2_-based nanomaterials, thereby expanding their potential for antimicrobial and biomedical applications.

## 1. Introduction

Titanium dioxide (TiO_2_) is among the most extensively studied semiconductor materials because of its chemical stability, low toxicity, and unique interfacial redox properties, making it attractive for applications in photocatalysis, antimicrobial systems, environmental remediation, and biomedicine [1,2]. However, its practical performance is limited by a wide bandgap (~3.2 eV for anatase), which restricts photoexcitation primarily to the UV region, and by rapid recombination of photogenerated charge carriers. Consequently, researchers have developed numerous strategies to extend the TiO_2_ photoresponse into the visible region and tailor its interfacial electronic and redox properties.

Among the various approaches, surface functionalization with naturally occurring organic ligands has emerged as an effective strategy for tailoring the interfacial electronic structure of TiO_2_ through interfacial charge-transfer (ICT) interactions [2,3,4,5,6,7,8,9]. Plant extracts contain chemically diverse molecules, including polyphenols and nitrogen-containing heterocyclic compounds, which possess distinct functional groups that can interact with Ti surface sites. Catechol- and galloyl-containing polyphenols readily coordinate to TiO_2_ through surface hydroxyl groups, forming Ti–O–C linkages that introduce donor states within the bandgap and promote visible-light absorption [2,9,10,11]. In parallel, nitrogen-containing molecules such as caffeine may also strongly interact with TiO_2_ through coordination involving heterocyclic nitrogen atoms, providing an alternative binding motif that contributes to the stabilization of the organic–inorganic interface while influencing its electronic structure [12]. Surface-bound organic ligands influence TiO_2_ not only by extending its optical response but also by modifying interfacial redox processes. Depending on their chemical structure, adsorbed molecules may alter charge separation, electron-transfer pathways, and the lifetimes of reactive intermediates generated upon irradiation [6,11,13]. Consequently, different classes of surface ligands may exert distinct effects on radical formation and scavenging, thereby reshaping the overall redox behavior of the semiconductor. Understanding how chemically diverse natural ligands influence these interfacial processes is therefore essential for rationally designing multifunctional TiO_2_-based materials.

Among the strategies employed to further optimize TiO_2_ performance, silver nanoparticles have attracted considerable attention for their ability to promote charge separation, facilitate electron transfer, and enhance the generation of reactive oxygen species [14,15,16]. In addition to their well-established antimicrobial activity, Ag nanoparticles exhibit plasmonic properties arising from localized surface plasmon resonance (LSPR), which can enhance visible-light absorption and modify interfacial charge-transfer processes, thereby influencing charge-carrier dynamics and ROS generation in TiO_2_-based hybrid materials. Ag nanoparticles can also act synergistically with surface-bound organic ligands to further modify interfacial electron-transfer processes. This combined organic–inorganic surface engineering offers opportunities to tune optical properties, redox behavior, and biological performance simultaneously, although the interplay among natural ligands, plasmonic Ag nanoparticles, and TiO_2_ interfaces remains poorly understood.

Unlike conventional TiO_2_/polyphenol or TiO_2_/Ag systems, which are commonly investigated primarily in terms of enhanced photocatalytic or antimicrobial performance, the present study focuses on how the composition of a multicomponent bio-derived interface governs competing interfacial redox pathways [17,18]. By combining chromatographic analysis of green tea constituents interacting with the TiO_2_ surface with DFT analysis, optical characterization, and EPR monitoring of radical processes, we distinguish the complementary roles of the organic ligands and plasmonic Ag nanoparticles in controlling the TiO_2_ interface. In particular, the study demonstrates that strong surface binding and ICT activity do not necessarily originate from the same molecular constituent, while subsequent Ag incorporation shifts the ligand-modified interface from predominantly radical-scavenging toward enhanced oxidative behavior.

In this work, we therefore investigate the interfacial redox engineering of TiO_2_ using green tea waste extract (GT), followed by silver functionalization, and examine how these interfacial modifications translate into biological performance. Combined HPLC and DFT analyses reveal distinct roles for adsorbed green tea constituents, with caffeine forming the most stable surface complex and EGCG producing the strongest ICT-related electronic perturbation. EPR measurements further demonstrate that GT functionalization suppresses excessive radical generation, whereas silver incorporation partially restores oxidative pathways. These interfacial redox differences are reflected in light-responsive antimicrobial activity and distinct cellular ROS responses. Thus, the main distinction of the present approach lies in establishing a structure–interface–redox–biological relationship rather than solely enhancing TiO_2_ photocatalytic or antimicrobial performance.

## 2. Results and Discussion

### 2.1. HPLC and DFT Investigation of Green Tea Constituent Adsorption on TiO_2_

**Figure 1:**
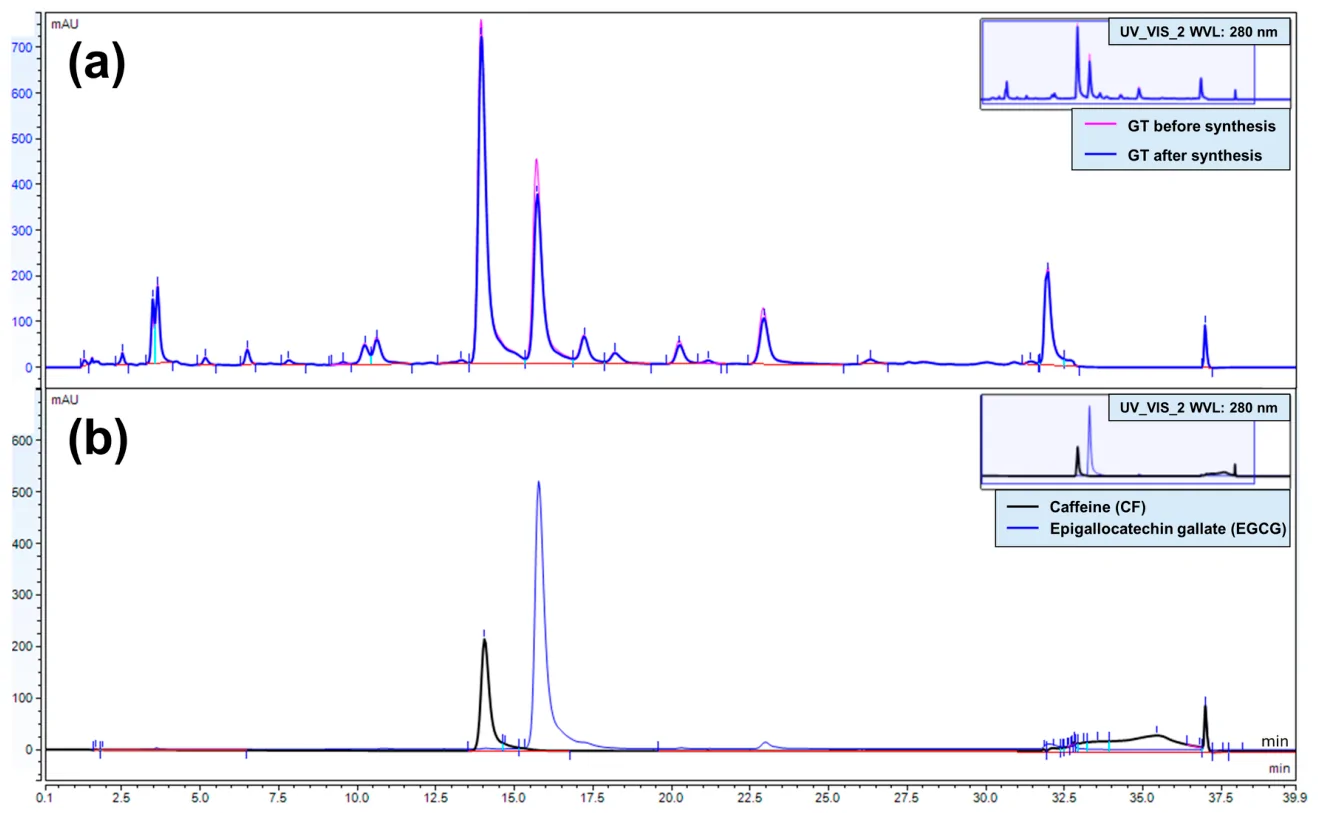
HPLC chromatograms recorded at 280 nm: (**a**) aqueous GT before and after ICT complex synthesis; (**b**) chromatograms of reference standards CF and EGCG acquired under identical chromatographic conditions.

High-performance liquid chromatography (HPLC) analysis confirmed the presence of multiple low-molecular-weight compounds in the aqueous extract of green tea waste material, in agreement with previously reported compositions of GT [19,20]. Representative chromatograms of the GT before and after contact with TiO_2_ are shown in Figure 1a. A clear decrease in the intensity of selected chromatographic peaks is observed after the synthesis step, indicating selective interaction between TiO_2_ and specific constituents of the green tea extract, consistent with the formation of ICT complexes [13].

To identify the compounds involved in this process, chromatograms of the reference standards CF and EGCG were recorded under identical chromatographic conditions (Figure 1b). Comparison of retention times revealed that the major peaks in the green tea extract correspond to CF (14.1 min) and EGCG (15.9 min). The marked reduction in the intensities of these peaks after TiO_2_ treatment indicates that these molecules preferentially interact with the oxide surface.

The selective decrease in CF and EGCG signals suggests their adsorption onto the TiO_2_ surface, consistent with the formation of surface-bound interfacial charge-transfer (ICT) complexes. Such behavior agrees with literature reports describing the coordination of polyphenolic and heterocyclic compounds to TiO_2_ via surface hydroxyl groups, leading to Ti–O–C linkages and related interfacial surface complexes [21,22]. HPLC analysis further revealed selective depletion of specific extract constituents after contact with TiO_2_, with EGCG showing the most pronounced decrease, indicating preferential interaction and chemisorption of redox-active molecules at the semiconductor surface.

The attenuation of HPLC peaks indicates that several green tea extract constituents interact with the TiO_2_ surface during nanocomposite synthesis. Among the monitored compounds, EGCG and caffeine showed the most pronounced decrease in peak intensity, indicating their involvement in the formation of the organic–inorganic interface. However, HPLC analysis alone does not provide information on the molecular origin, adsorption mode, or relative strength of these interactions. Therefore, DFT calculations were performed to elucidate the adsorption mechanisms of representative green tea constituents and their influence on the electronic structure of TiO_2_.

To gain molecular-level insight into the interactions suggested by the HPLC analysis, DFT calculations were performed using representative green tea constituents, EGCG and caffeine, adsorbed on the anatase TiO_2_ (101) surface. The calculations aimed to compare their preferred adsorption modes, relative binding strengths, and influence on the electronic structure of TiO_2_. In addition to optimized adsorption geometries, the density of states (DOS) and frontier molecular orbitals (FMOs) were analyzed to elucidate the relationship between molecular adsorption and the experimentally observed optical properties. DFT calculations predict that EGCG and caffeine interact with TiO_2_ through fundamentally different coordination mechanisms. The EGCG ligand coordinates to Ti atoms through deprotonated hydroxyl groups [13], whereas caffeine binds through the unmethylated nitrogen atom of the xanthine ring.

**Scheme 1:**
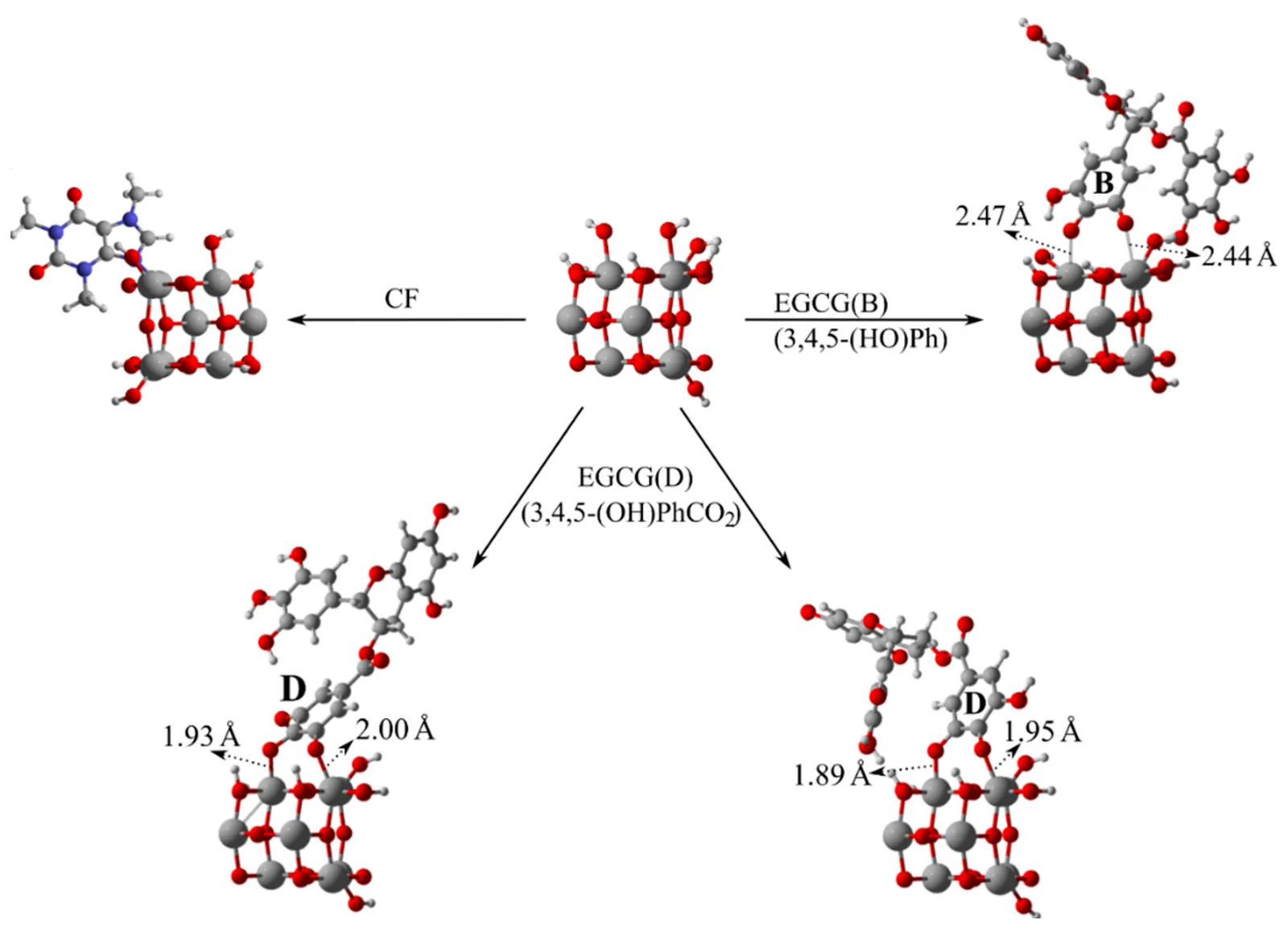
The optimized geometries of the TiO_2_-cluster and its adducts with molecules from green tea (EGCG and CF). (gray—carbon; white—hydrogen; red—oxygen; blue—nitrogen; large gray—titanium).

The EGCG ligand can be coordinated to TiO_2_ through the hydroxyl groups of the 3,4,5-trihydroxybenzoate group (ring D) or via the 3,4,5-trihydroxyphenyl group (ring B), as indicated in the literature [23]. Both coordinations result from a condensation reaction between the ligand’s hydroxyl groups and the TiO_2_ surface hydroxyl groups. The size of the EGCG ligand bends all these geometries, enabling additional hydrogen bonding (Scheme 1). When the EGCG molecule is bound to the TiO_2_ cluster via the hydroxyl groups of the 3,4,5-trihydroxybenzoate ring (D), the length of Ti-O bonds spans from 1.93 to 2.00 Å. On the other hand, coordination via the hydroxyl groups of the 3,4,5-trihydroxyphenyl ring results in longer Ti-O bonds (2.44 and 2.47 Å). The adsorption of CF causes a slightly distorted geometry with respect to the 101 crystal plane of TiO_2_, with a Ti-N bond of 2.26 Å.

**Figure 2:**
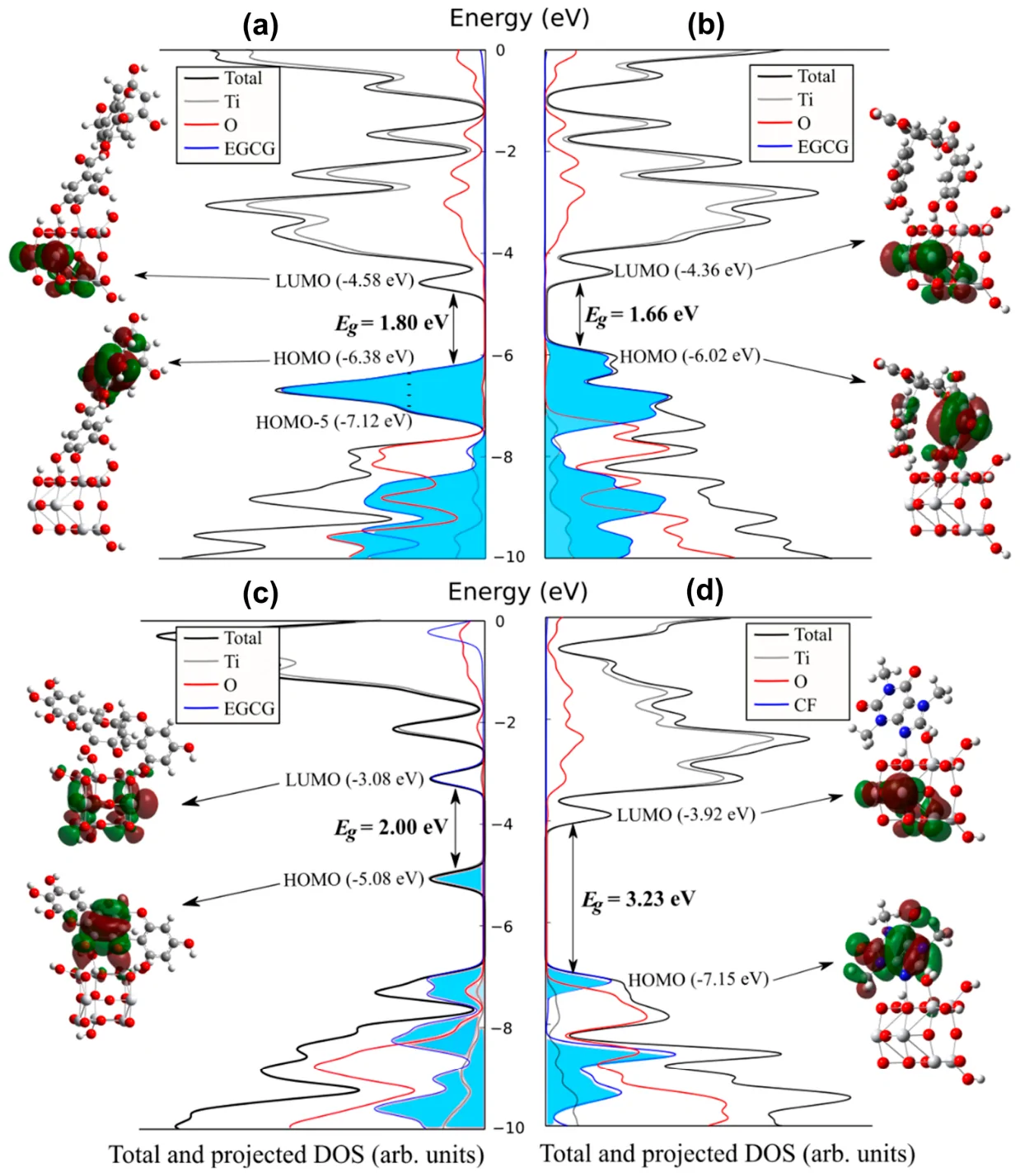
The total and partial density of states (TDOS/PDOS) diagrams of (**a**,**b**) EGCG(D)/[Ti_8_O_12_(OH)_6_]; (**c**) EGCG(B)/[Ti_8_O_12_(OH)_6_]; and (**d**) CF/[Ti_8_O_12_(OH)_8_] clusters. (gray—carbon; white—hydrogen; red—oxygen; blue—nitrogen; large gray—titanium).

**Figure 3:**
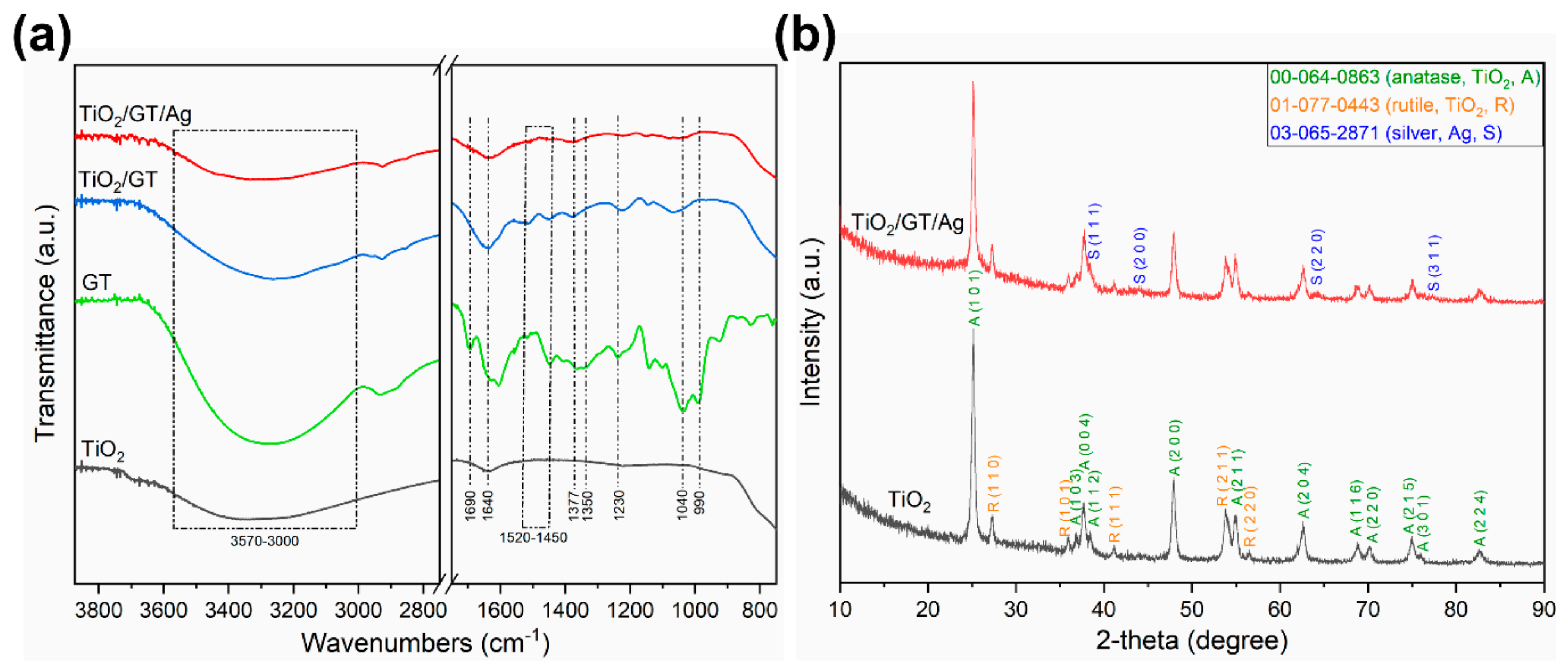
(**a**) FTIR spectra of pristine TiO_2_, green tea extract (GT), TiO_2_/GT, and TiO_2_/GT/Ag nanocomposites and (**b**) XRD patterns of pristine TiO_2_ and TiO_2_/GT/Ag nanocomposite. The indexed reflections correspond to anatase TiO_2_ (A), rutile TiO_2_ (R), and metallic silver (S).

**Figure 4:**
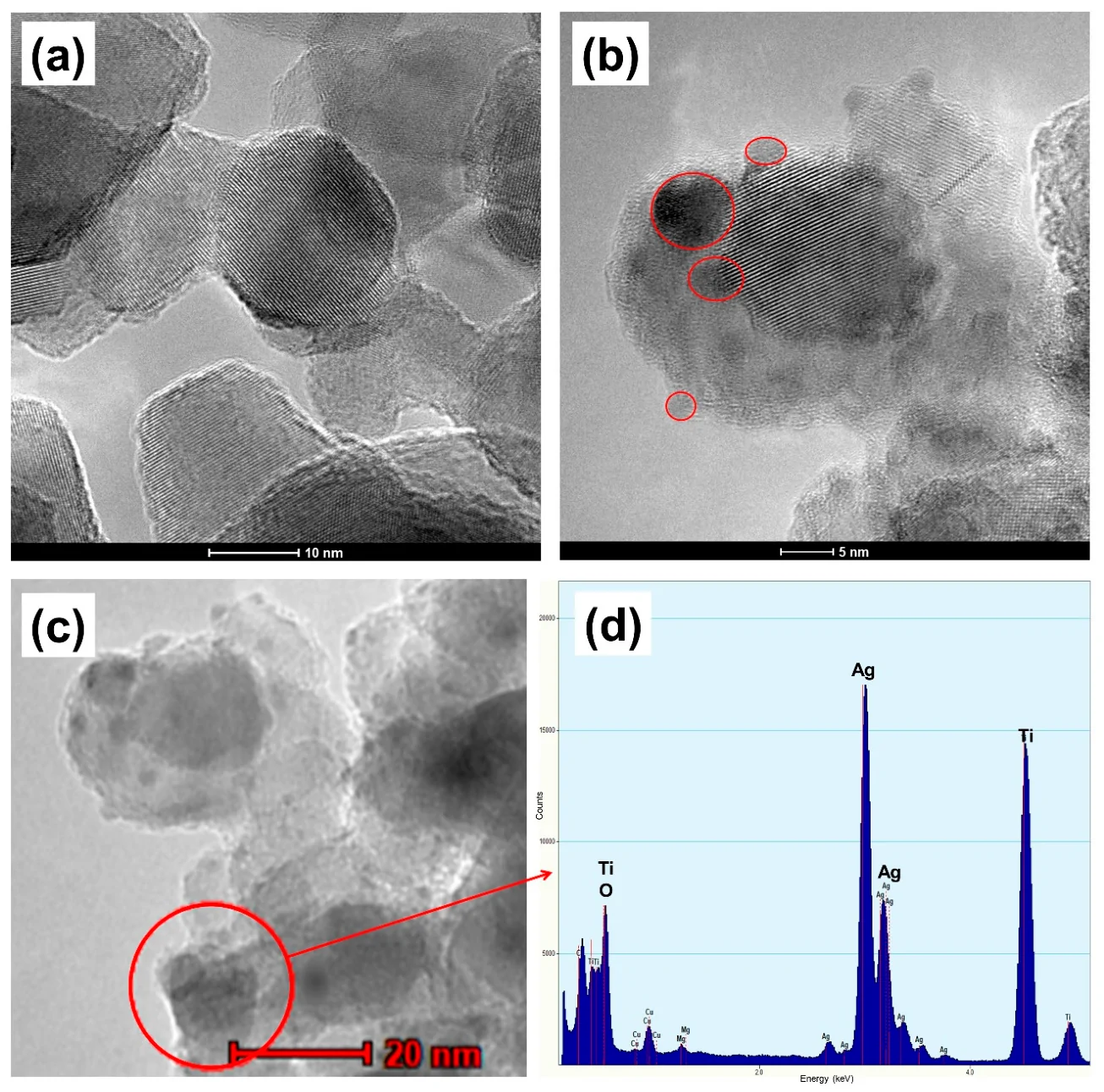
TEM images of (**a**) TiO_2_/GT and (**b**,**c**) TiO_2_/GT/Ag nanocomposites, showing predominantly spherical Ag nanoparticles (~2–5 nm) distributed on the TiO_2_/GT surface; (**d**) EDS spectrum acquired from the selected region in (**c**), confirming the presence of Ag.

**Figure 5:**
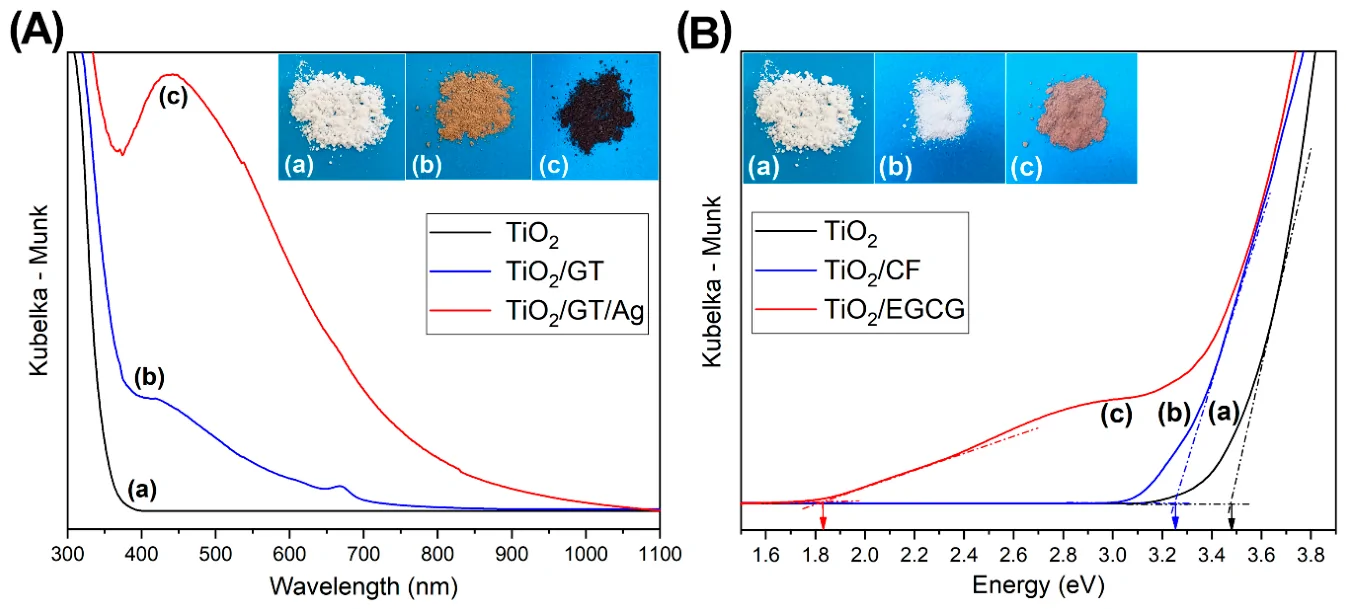
DRS analysis of pristine TiO_2_ and TiO_2_-based nanocomposites: (**A**) Kubelka–Munk-transformed DRS spectra of (a) TiO_2_, (b) TiO_2_/GT, and (c) TiO_2_/GT/Ag nanocomposites, with inset photographs showing the corresponding powder colors; (**B**) Kubelka–Munk plots used for bandgap estimation of pristine (a) TiO_2_ and TiO_2_ nanocomposites synthesized with individual GT compounds (b) CF and (c) EGCG).

The total and partial density of states (TDOS/PDOS) diagrams for the EGCG/[Ti_8_O_12_(OH)_6_] and CF/[Ti_8_O_14_(OH)_4_] clusters with the corresponding HOMO/LUMO molecular orbitals are displayed in Figure 2. We present the DOS diagram for the EGCG(D)/[Ti_8_O_12_(OH)_6_] complexes in Figure 2a,b. In contrast, the DOS diagram for the EGCG(B)/[Ti_8_O_12_(OH)_6_] is shown in Figure 2c. The HOMO orbitals of EGCG(D)/[Ti_8_O_12_(OH)_6_] complexes consist of the π-orbitals of benzopyran-3-yl (Figure 2a) and 3,4,5-trihydroxybenzoate (Figure 2b) aromatic rings. These orbitals serve as donor levels and lie within the TiO_2_ bandgap. On the other hand, the HOMO level of the CF/[Ti_8_O_14_(OH)_4_] cluster is spread across the entire xanthine ring and is located at the edge of the VB (Figure 2d). In all cases, the valence bands (VB) are primarily distributed over the oxygen atoms of the clusters (2p orbitals). In contrast, the conduction bands (CB) are mainly formed from the empty 3d orbitals of the Ti atoms in the clusters. The position of CBMs is approximately −4 eV, which aligns with experimental results [23]. However, because the orbitals of surface hydroxyl groups and lattice oxygen atoms mix, it is impossible to determine the exact location of VBM. As a result, the bandgap values of the TiO_2_ cluster in these systems remain uncertain. The donor levels of surface-modified TiO_2_, which correlate with the ligands’ HOMO orbitals, lie within the bandgap, as previously noted. For the two conformations of the EGCG(D)/[Ti_8_O_12_(OH)_6_] cluster, these levels are set at potentials of −6.38 and −6.02 eV, resulting in Δ^HOMO-LUMO^ gaps of 1.80 and 1.66 eV, respectively (Figure 2a,b). The HOMO-LUMO gaps are in reasonable agreement with the experimental optical band gaps, although the optical absorption arises from the excited-state transitions. These values are close to the onset of the absorption of EGCG/TiO_2_ nanopowder (1.83 eV; Figure 5). A slightly higher value than the experimentally determined optical bandgap is obtained with the EGCG(B)/[Ti_8_O_12_(OH)_6_] cluster (Δ^HOMO-LUMO^ = 2.00 eV; Figure 2c); however, this complex does not accurately reproduce the electronic structure of the TiO_2_ cluster, as it overestimates the CBM position by 1 eV. The likely reason is the loose coordination of EGCG via ring B, which is in diketone form and is generally less stable than the enol form. In contrast, the excellent agreement between experimentally determined and theoretically predicted bandgap is achieved for the CF/[Ti_8_O_14_(OH)_4_], with the HOMO-LUMO gap only 0.09 eV overestimated as compared to the optical bandgap (3.23 vs. 3.25 eV; Figure 2d and Figure 5). Although both EGCG and caffeine adsorb onto the TiO_2_ surface, their electronic effects differ substantially. EGCG introduces donor states within the TiO_2_ bandgap that facilitate ligand-to-semiconductor charge-transfer transitions, whereas caffeine preserves TiO_2’_s intrinsic electronic structure despite its stronger thermodynamic binding. These findings demonstrate that adsorption strength alone does not determine the electronic response of the hybrid interface.

The computed formation energies of two isomers of the EGCG(D)/[Ti_8_O_12_(OH)_6_] complex are comparable: −11.50 and −9.41 kcal/mol. The isomer EGCG(B)/[Ti_8_O_12_(OH)_6_] has a somewhat higher formation energy of −15.42 kcal/mol. These differences in the formation energies of ICT complexes between the EGCG molecule and the TiO_2_ cluster are mainly due to intermolecular hydrogen bonding. In contrast, caffeine binds much more strongly to the TiO_2_ cluster, with a calculated formation energy of −34.55 kcal/mol. Unlike EGCG, whose hydroxyl groups must be deprotonated prior to coordination, caffeine is a neutral Lewis base that coordinates directly to surface Ti atoms through the unmethylated nitrogen atom, accounting for its substantially stronger binding. These results provide a molecular explanation for the HPLC observations, indicating that multiple green tea constituents adsorb on the surface through distinct coordination mechanisms. Importantly, although caffeine forms the thermodynamically most stable surface complex, EGCG exhibits much stronger electronic coupling with TiO_2_, giving rise to ICT states responsible for the experimentally observed visible-light absorption. These findings demonstrate that adsorption strength and electronic perturbation of the semiconductor are complementary, rather than equivalent, characteristics of the organic-TiO_2_ interface.

### 2.2. Structural and Morphological Characterization of the Surface-Modified TiO_2_ Nanocomposites

The FTIR spectra of pristine TiO_2_, green tea extract (GT), TiO_2_/GT, and TiO_2_/GT/Ag nanocomposites are presented in Figure 3a. The broad absorption band observed in the 3000–3570 cm^−1^ region, attributed to O–H stretching vibrations [1,24], is prominent in the GT spectrum and gradually decreases in intensity upon nanocomposite formation. This attenuation indicates that hydroxyl groups interact with the TiO_2_ surface, consistent with ligand chemisorption, condensation reactions, and the formation of interfacial Ti–O–C linkages. Distinct absorption bands observed at approximately 1690 cm^−1^ and 1640 cm^−1^ in the GT spectrum are assigned to C=N and C=C stretching vibrations of the purine ring in caffeine [25]. The disappearance of these bands is consistent with DFT calculations indicating coordination of caffeine to surface Ti atoms through the unmethylated nitrogen atom. Additional bands appearing at 1377 cm^−1^ and 1350 cm^−1^ are attributed to C–O–C and C–O stretching vibrations characteristic of phenolic compounds [26], while the broad absorptions centered around 1230, 1040, and 990 cm^−1^ correspond to hydroxyl-containing groups within the gallate moiety of EGCG [27]. Changes in the intensity and definition of these bands after nanocomposite formation further support the participation of multiple green tea constituents in forming the organic–inorganic interface. In the region between 1450 and 1520 cm^−1^, absorption features associated with CH_2_ bending vibrations, carboxylate (COO^−^) groups, and aromatic C–H deformations are observed, reflecting organic components derived from GT [27]. Upon nanocomposite formation, these bands exhibit changes in intensity and definition, further supporting the selective binding of extract constituents to the TiO_2_ surface. In the TiO_2_/GT/Ag sample, a further decrease in the intensity of the O–H stretching band around 3000 cm^−1^ is observed, indicating the involvement of hydroxyl groups during silver functionalization. Overall, the FTIR results confirm successful surface functionalization of TiO_2_ with green tea-derived ligands, consistent with the HPLC and DFT analyses, while preserving the chemical integrity of the hybrid material.

The X-ray diffraction patterns of pristine TiO_2_ and the TiO_2_/GT/Ag nanocomposite are shown in Figure 3b. The diffractogram of commercial TiO_2_ exhibits characteristic reflections corresponding to a mixed anatase-rutile phase composition. The diffraction peaks at 2θ = 25.3° (101), 37.9° (004), and 48.0° (200) are assigned to the anatase phase, while the peaks at 27.3° (110), 35.9° (101), and 54.1° (211) correspond to the rutile phase. These reflections agree well with standard reference data for TiO_2_ and confirm the starting material’s crystalline structure. In the TiO_2_/GT/Ag nanocomposite, the characteristic TiO_2_ diffraction peaks are preserved, indicating that the crystalline framework of the oxide remains unchanged after surface modification. In addition, new diffraction peaks appear at 2θ = 38.1° (111), 44.3° (200), and 77.4° (311), which can be assigned to the face-centered cubic structure of metallic silver. The preservation of the TiO_2_ crystal structure, along with the appearance of characteristic Ag reflections, shows that surface functionalization and silver incorporation occur without disrupting the crystalline oxide framework. These structural findings provide a solid basis for interpreting the subsequent changes in the electronic, redox, and biological properties of the nanocomposites.

Representative TEM micrographs of pristine TiO_2_ are shown in Figure 4a. The sample consists of nearly spherical to irregularly rounded nanoparticles with an average size of approximately 15 nm, along with a population of smaller particles in the ~10 nm range. The sample shows moderate aggregation, characteristic of metal oxide nanoparticles with high surface energy. In contrast, the TiO_2_-based nanohybrid exhibits a clearly modified surface morphology. As shown in Figure 4b,c, the TiO_2_ particles are decorated with numerous nanoscale features appearing as darker protrusions on the oxide surface. These features are attributed to predominantly spherical silver nanoparticles, approximately 2–5 nm in size, confirming their successful deposition onto the TiO_2_/GT matrix. The relatively uniform distribution of these nanoparticles suggests that the green tea-derived surface functionalities help anchor and stabilize the metallic species. Energy-dispersive X-ray spectroscopy (EDS) analysis of the TiO_2_/GT/Ag nanocomposite (Figure 4d) confirms the presence of Ti and O as the main constituents of the oxide matrix, together with distinct Ag signals corresponding to the silver nanoparticles observed in the TEM images. A moderate carbon signal originates from the organic components of the green tea extract. Minor magnesium contributions may arise from trace impurities, while copper signals are attributed to the TEM grid and are not associated with the nanocomposite itself. Overall, the combined TEM and EDS analyses provide direct morphological and elemental evidence of successful formation of the TiO_2_/GT/Ag nanohybrids, complementing the structural information from FTIR and XRD.

ICP analysis confirmed successful silver incorporation, with a Ag content of 5.1 wt% relative to the total mass of the TiO_2_/GT/Ag nanocomposite. This result demonstrates efficient silver loading onto the functionalized TiO_2_ surface and provides a quantitative basis for interpreting the material’s subsequent optical, redox, and biological properties.

### 2.3. Optical Properties of Surface-Modified TiO_2_ Nanocomposites

The optical properties of pristine TiO_2_ and the corresponding nanocomposites were investigated by DRS. As shown in Figure 5A, surface modification of TiO_2_ with green tea-derived compounds produces a pronounced change in the material’s optical response, accompanied by a visible color change in the powders. These observations are consistent with surface condensation reactions between TiO_2_ hydroxyl groups and functional groups on the extract components, forming Ti–O–C linkages. A comparable color change is also observed after subsequent surface functionalization with Ag nanoparticles. The DRS reveals a significant red shift of the absorption edge upon surface modification, indicating the emergence of new electronic states within the TiO_2_ bandgap. The observed red shift and reduced apparent bandgap suggest the formation of interfacial electronic states associated with ligand-to-semiconductor charge-transfer interactions, which may influence both visible-light absorption and interfacial radical processes. Such spectral changes are characteristic of surface-bound ICT complexes. Notably, the TiO_2_/GT/Ag nanocomposite exhibits a broad absorption feature centered at ~440 nm, attributed to the localized surface plasmon resonance of Ag nanoparticles, further confirming their successful incorporation.

To elucidate the contribution of individual green tea constituents to the observed optical behavior, TiO_2_ nanocomposites were synthesized separately from CF and EGCG, as identified by HPLC analysis. The corresponding Kubelka–Munk plots and Tauc extrapolations (Figure 5B) reveal a substantial narrowing of the apparent bandgap for TiO_2_/EGCG, yielding an apparent bandgap of approximately 1.83 eV. In contrast, TiO_2_/CF displays a significantly smaller shift, with an estimated bandgap of ~3.25 eV, close to that of pristine TiO_2_ (~3.48 eV). These results indicate that polyphenolic compounds with multiple hydroxyl groups (such as EGCG) are primarily responsible for strong electronic coupling to TiO_2_ and the formation of effective ICT states, whereas CF exhibits a considerably weaker effect. The DRS analysis thus provides direct spectroscopic evidence that the optical and electronic properties of TiO_2_ are selectively modified by specific GT-derived molecules, in full agreement with the HPLC and FTIR findings. The optical response of the nanocomposites is therefore governed not only by surface adsorption but also by the nature of the adsorbed ligand. While both EGCG and caffeine contribute to the formation of the organic–inorganic interface, only EGCG induces a significant electronic perturbation of TiO_2_ via ICT, resulting in the experimentally observed visible-light absorption.

### 2.4. EPR Investigation of Charge Carrier Dynamics and Radical Processes

**Figure 6:**
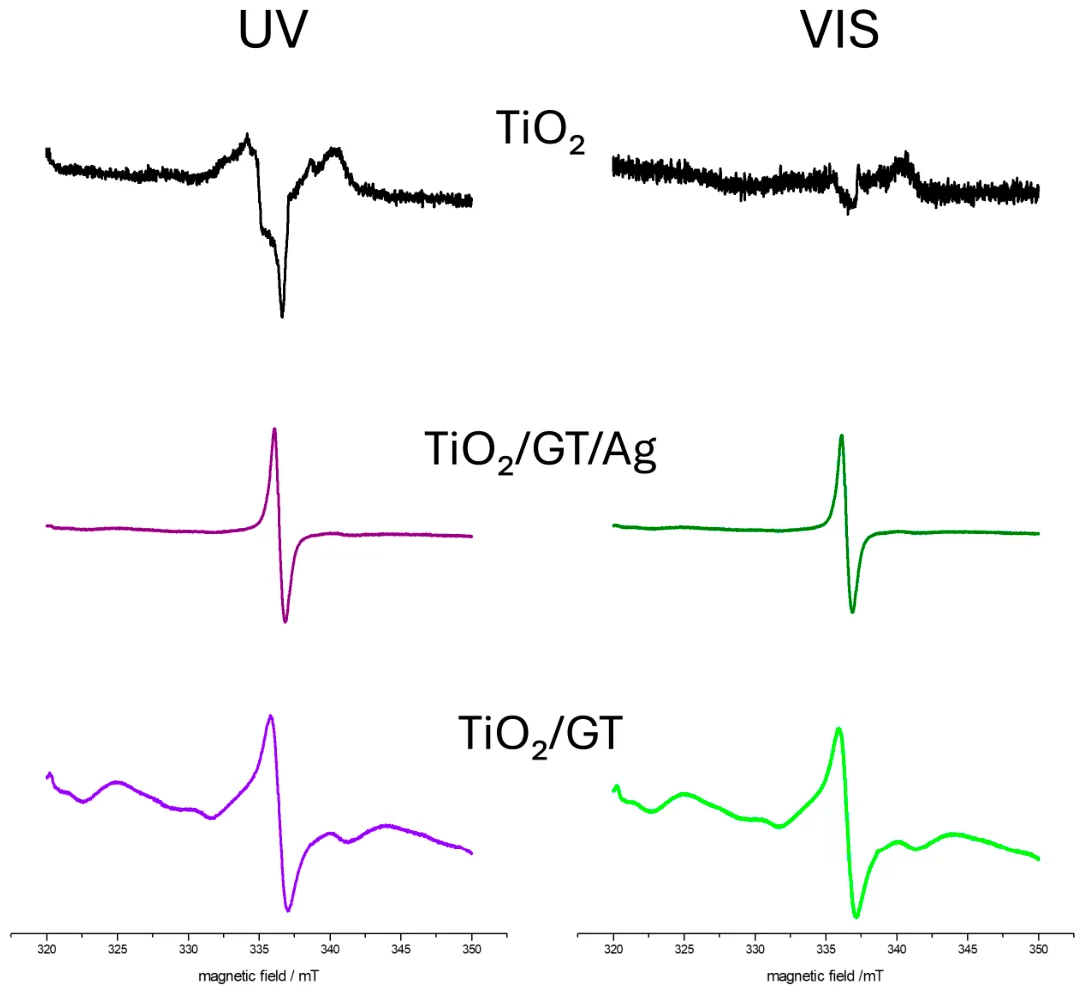
The EPR spectra of pristine and green tea-modified TiO_2_ (GT or GT/Ag), measured in the solid state upon UV (LED@365 nm, (**left**)) and VIS (>420 nm, (**right**)) at 100 K.

Solid-state EPR spectra of powdered nanocomposites acquired at 100 K are shown in Figure 6. No significant signals were visible in the dark. Upon UV irradiation, pristine TiO_2_ exhibited signals characteristic of intrinsic paramagnetic centers [28]. In contrast, the GT-modified samples showed markedly different EPR responses after irradiation, indicating altered charge-carrier behavior induced by surface functionalization. These signals are consistent with the formation of stabilized surface-associated radical species originating from interactions between chemisorbed organic molecules and the TiO_2_ surface. Broad contributions observed in the TiO_2_/GT sample are attributed to trace transition-metal ions, such as Mn^2+^ (Figure S2, third line), which are also detected in the EPR spectrum of the aqueous suspensions of pure extract (Figure S2), and their presence is typical in the natural material used for the modification [29]. Under VIS light irradiation, analogous signals were recorded, although with slightly lower intensity.

**Figure 7:**
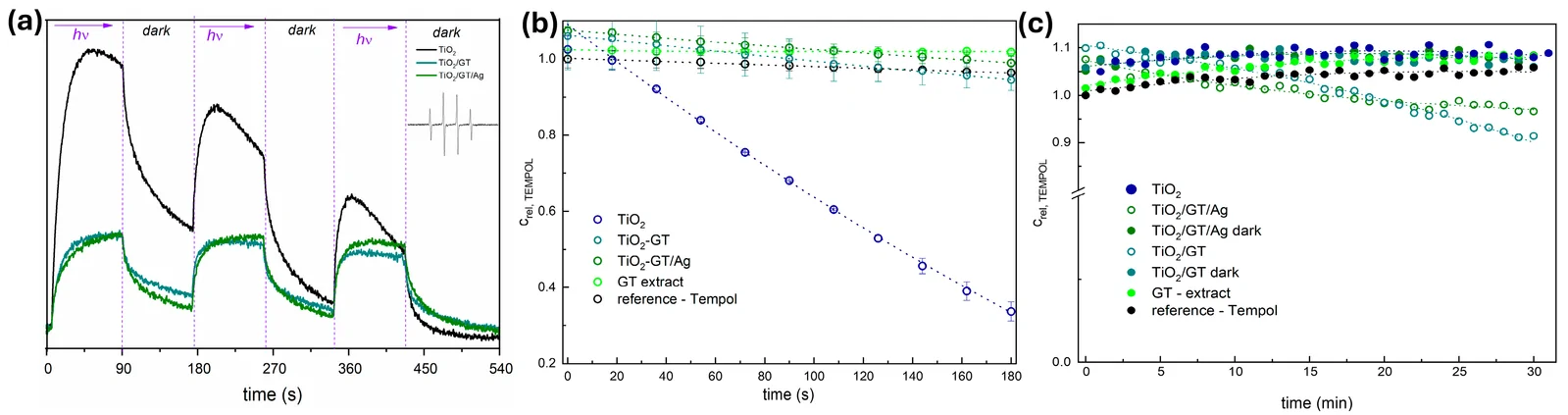
Time dependence of (**a**) EPR signal intensity for ^•^DMPO-OH spin adduct (c_0,DMPO_ = 0.04 M) and (**b**,**c**) TEMPOL relative concentration (c_0,TEMPOL_ = 25 µM) monitored in aqueous aerated suspensions of pristine, modified TiO_2_ (GT or GT/Ag; photocatalyst load 0.25 mg/mL), and GT extract (0.25 mg/mL) under UV (365 nm, (**b**)) and visible (>420 nm, (**c**)) irradiation. The symbols represent the experimental data, while the dotted lines show their least-squares fits. Note: TiO_2_ and GT extract in the dark are omitted for clarity—identical curve as under visible irradiation.

Because solid-state measurements cannot identify transient species, we performed indirect EPR measurements using spin-trapping techniques to investigate radical formation in aqueous suspensions. Upon UV irradiation, characteristic •DMPO-OH signals were detected for all samples (Figure 7a, inset), confirming the formation of reactive species. Time-dependent measurements revealed a rapid increase in signal intensity, followed by decay upon cessation of irradiation, reflecting the limited stability of the spin adduct and likely the rapid recombination of photogenerated charge carriers in the dark. The markedly lower spin-adduct intensities observed for GT-modified samples indicate suppression of excessive radical generation by green tea-derived surface ligands [30]. A comparable photoresponse pattern was observed with the POBN spin trap (Figure S3), with a particularly low signal intensity for the TiO_2_/GT/Ag sample.

Stable nitroxide radicals bearing a paramagnetic NO• group, such as Tempo derivatives, offer an alternative indirect EPR approach to probing radical processes in photocatalytic systems (Figure 7b). These Tempo derivatives, including 4-hydroxy-2,2,6,6-tetramethylpiperidin-1-oxyl (Tempol) with a characteristic three-line EPR spectrum, readily react with photogenerated reactive species via their nitroxide moiety, yielding diamagnetic products and enabling assessment of the overall radical-scavenging capacity of the investigated materials [13]. The strongest TEMPOL depletion under UV irradiation was observed for pristine TiO_2_, confirming its highest radical-generating activity. Surface functionalization with GT markedly suppressed this activity, whereas Ag incorporation partially restored oxidative pathways compared with TiO_2_/GT.

The complementary oxidative pathway was examined using sterically hindered amine TMPO, which is converted to the corresponding paramagnetic nitroxide form (Tempone) upon oxidation (Figure S4). The most pronounced Tempone signal was observed for pristine TiO_2_ under UV irradiation, as expected for an unmodified photocatalyst. Functionalized samples exhibited weaker oxidation responses, suggesting that surface-bound organic ligands partially scavenge photogenerated radicals, thereby reducing oxidative capacity. Nevertheless, silver enhances oxidation relative to TiO_2_/GT, consistent with its role in modulating the system’s redox balance.

These findings demonstrate that surface functionalization does not merely enhance or suppress photoinduced radical generation but rather redirects interfacial redox pathways, depending on the nature of the surface species.

**Figure 8:**
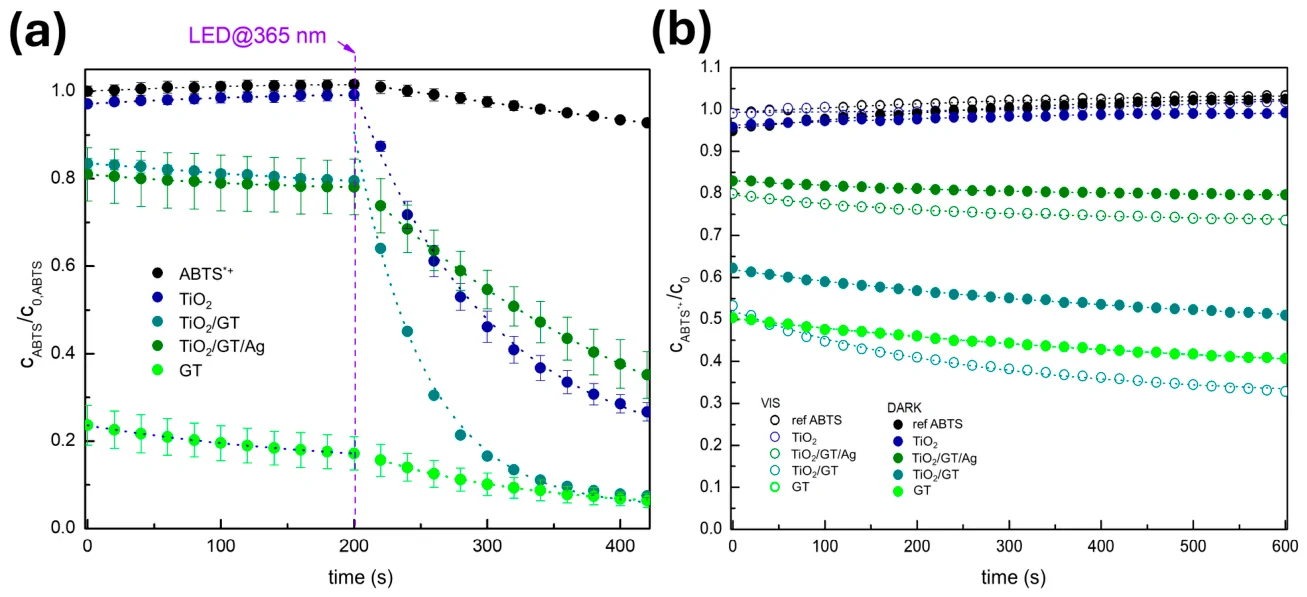
(**a**) Time dependence of relative ABTS^•+^ concentration monitored by EPR in aqueous aerated suspensions of pristine, modified TiO_2_ (GT or GT/Ag; photocatalyst load 0.25 mg/mL), and (**b**) GT extract (0.025 mg/mL) in the dark and under UV (365 nm) or visible (>420 nm) irradiation. The symbols represent the experimental data, while the dotted lines show their least-squares fits.

EPR experiments utilizing the semi-stable radical cation (ABTS^•+^) supplement the investigation of the photoactivity and radical-scavenging properties of TiO_2_ functionalized with green tea extract. The ABTS^•+^ radical cation exhibits a characteristic single-line EPR signal in overmodulated mode, which progressively decreases as it is reduced to its diamagnetic parent compound. This signal decay reflects two concurrent processes: (i) chemical reduction of ABTS^•+^ by antioxidant compounds [31], present in the green tea extract immobilized on the TiO_2_ surface; and (ii) photoreduction driven by electrons generated upon irradiation of the TiO_2_ photocatalyst [13]. The observed reduction kinetics provide insight into the dual mechanism of the green tea-modified TiO_2_ system. As expected, green tea-derived ligands immobilized on the TiO_2_ surface contribute to radical elimination, consistent with the well-established structure-dependent radical-scavenging properties of tea polyphenols. Computational studies have further shown that the antioxidant activity of tea-derived polyphenols is strongly governed by their molecular conformation and favorable hydrogen-atom-transfer pathways [32]. However, silver particles appear to suppress this antioxidant activity (Figure 8). While UV light exposure enhanced the elimination of ABTS^•+^ in all systems with pristine or modified TiO_2_, most likely via the generation of photoelectrons in both modified samples, VIS light significantly improved the reduction/elimination of ABTS^•+^ only in the sample without silver.

To evaluate radical-scavenging properties and phototoxicity, the DPPH assay was employed. A decrease in the DPPH radical signal (Figure S5) reflects effective radical scavenging, suggesting that surface modification influences both generation and quenching dynamics. As expected, green tea-derived ligands immobilized on the TiO_2_ surface contribute to radical elimination. While green tea-derived surface ligands are expected to dominate this antioxidant response, caffeine adsorption, as shown by HPLC and DFT, may also contribute to the overall redox behavior of the hybrid interface. Importantly, the TiO_2_/GT and TiO_2_/GT/Ag samples retained this scavenging ability, demonstrating that surface modification preserved the extract’s antioxidant functionality. The initial rapid signal drop in extract-containing systems resulted from the rapid reaction between DPPH and green tea-derived surface ligands. UV irradiation further enhanced radical termination in all samples, whereas visible-light induced only minor changes, though it still affected DPPH decay kinetics (Figure S6).

### 2.5. Antimicrobial Activity

**Figure 9:**
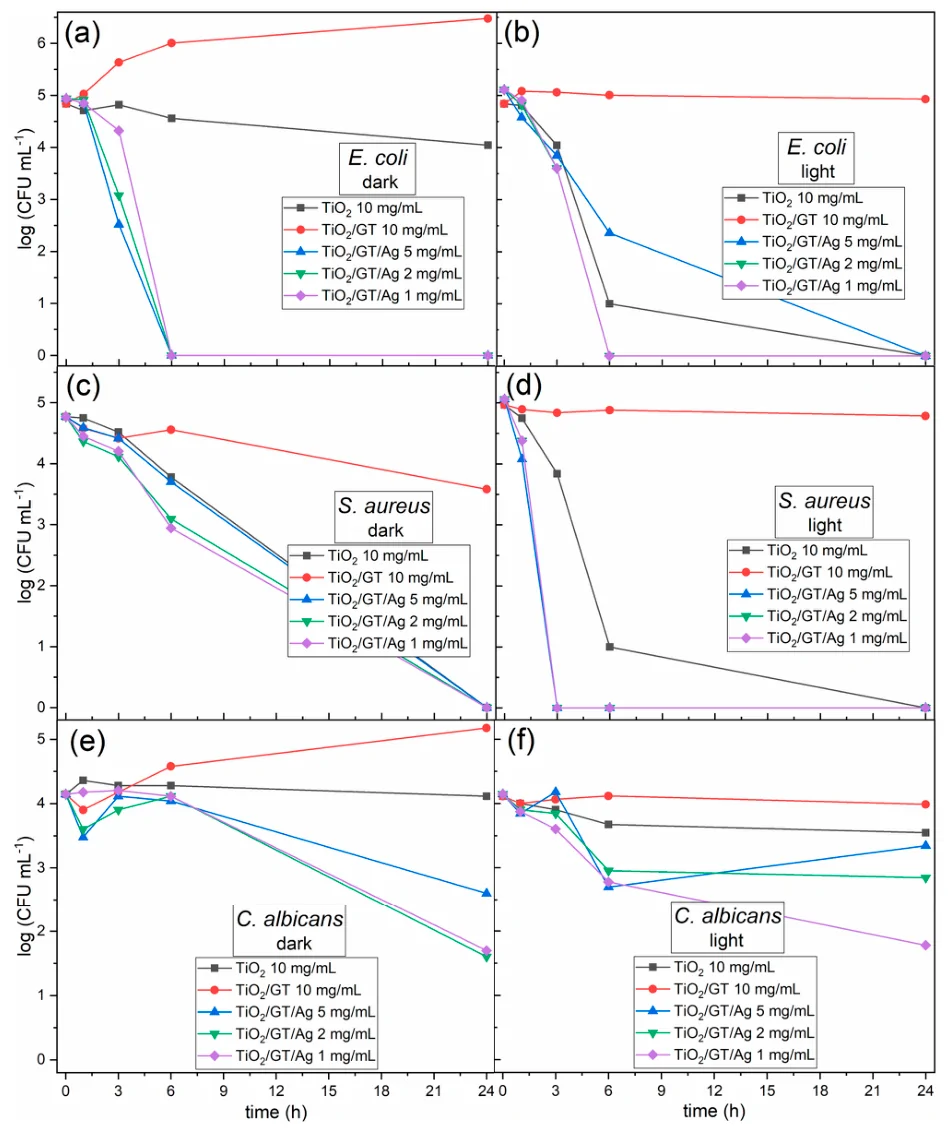
Antimicrobial activity of pristine TiO_2_, TiO_2_/GT, and TiO_2_/GT/Ag nanocomposites against *Escherichia coli*, *Staphylococcus aureus*, and *Candida albicans* evaluated under dark conditions (**a**,**c**,**e**), and upon visible-light irradiation (436 nm) (**b**,**d**,**f**).

The antimicrobial activity of pristine TiO_2_, TiO_2_/GT, and TiO_2_/GT/Ag nanocomposites was evaluated against *Escherichia coli*, *Staphylococcus aureus*, and *Candida albicans* under dark conditions and upon visible-light irradiation (Figure 9). The antimicrobial activity closely follows the interfacial redox behavior established by the EPR experiments, demonstrating that microbial inactivation depends on both the surface chemistry of the nanocomposites and the susceptibility of the target microorganism.

Among the investigated systems, TiO_2_/GT/Ag exhibited the highest antimicrobial efficiency against all tested microorganisms. ICP analysis confirmed successful silver incorporation, with a Ag content of 5.1 wt% relative to the total mass of the nanocomposite, providing a quantitative basis for interpreting the contribution of silver to the observed antimicrobial performance. This selective light response indicates that visible-light-induced oxidative pathways contribute more efficiently to microbial inactivation in the Gram-positive system. Notably, visible-light irradiation led to a pronounced additional enhancement of antimicrobial activity exclusively against *S. aureus*, whereas for *E. coli* and *C. albicans*, the differences between dark and irradiated conditions were marginal, with slightly stronger inhibition observed in the dark in some cases. The light-enhanced antimicrobial response observed for *S. aureus* can be attributed to the synergistic action of photogenerated reactive oxygen species (ROS) and silver-related mechanisms. As a Gram-positive bacterium, *S. aureus* lacks an outer membrane, thereby facilitating direct interaction between ROS and the cytoplasmic membrane and intracellular targets. Consequently, photoinduced oxidative stress generated at the TiO_2_/GT/Ag surface under visible-light irradiation can more efficiently compromise cell integrity, thereby enhancing bacterial inactivation [33,34,35,36,37]. In contrast, Gram-negative *E. coli* has an additional outer membrane composed of lipopolysaccharide, which can act as a diffusion barrier for ROS and reduce the impact of photoactivated processes [1]. Similarly, the thick, complex cell wall of *C. albicans*, composed of glucans, mannoproteins, and chitin, together with its efficient antioxidant defense mechanisms, likely attenuates the contribution of light-induced ROS, resulting in comparable antimicrobial effects under both dark and illuminated conditions. Interestingly, for *E. coli* and *C. albicans*, antimicrobial effects under dark conditions were comparable to or slightly stronger than those under visible-light irradiation. Considering the Ag loading determined by ICP (5.1 wt%), this behavior suggests that light-independent mechanisms associated with incorporated silver species make a substantial contribution to microbial inactivation in these organisms. In aqueous environments, silver ions released from the nanocomposite interact with thiol-containing proteins, enzymes, and membrane components, leading to cellular dysfunction even in the absence of light [35]. Under visible-light irradiation, the contribution of photocatalytically generated ROS becomes more pronounced, thereby modifying the relative contribution of silver-mediated and photoinduced antimicrobial pathways. Taken together with the ICP-determined Ag loading (5.1 wt%), these findings indicate that the antimicrobial response reflects the balance between incorporated silver species and interfacial oxidative/antioxidant pathways rather than solely the efficiency of photocatalytic ROS generation.

Pristine TiO_2_ showed moderate antimicrobial activity, which increased under visible-light irradiation, consistent with photocatalytic ROS generation. This effect was observed across all tested strains, confirming that photoactivated TiO_2_ can induce sufficient oxidative stress to impair microbial viability [37]. In contrast, TiO_2_/GT exhibited little or no antimicrobial activity under either dark or visible-light conditions. This behavior is fully consistent with the EPR results, which demonstrated pronounced radical-scavenging activity of the green tea-derived surface ligands. HPLC and DFT analyses further showed that the organic interface consists of strongly adsorbed green tea-derived ligands, with EGCG introducing ICT states and caffeine forming stable surface complexes. Rather than promoting oxidative stress, these surface-bound ligands collectively favor radical-scavenging processes, thereby preventing efficient microbial inactivation. A possible additional contribution may arise from residual organic components that could support microbial adaptation or growth under nutrient-limited conditions [38,39].

Overall, the antimicrobial performance of the nanocomposites is governed by the interplay between interfacial electronic structure, redox processes, and silver-mediated antimicrobial mechanisms. Surface functionalization with green tea-derived ligands shifts the TiO_2_ interface toward radical scavenging, whereas subsequent silver incorporation partially restores oxidative pathways, enhancing antimicrobial activity. The pronounced visible-light response observed for *S. aureus* further shows that biological performance depends not only on the presence of silver but also on the interfacial redox properties established through organic–inorganic surface engineering.

### 2.6. Cytotoxicity and Oxidative Stress Assessment

**Figure 10:**
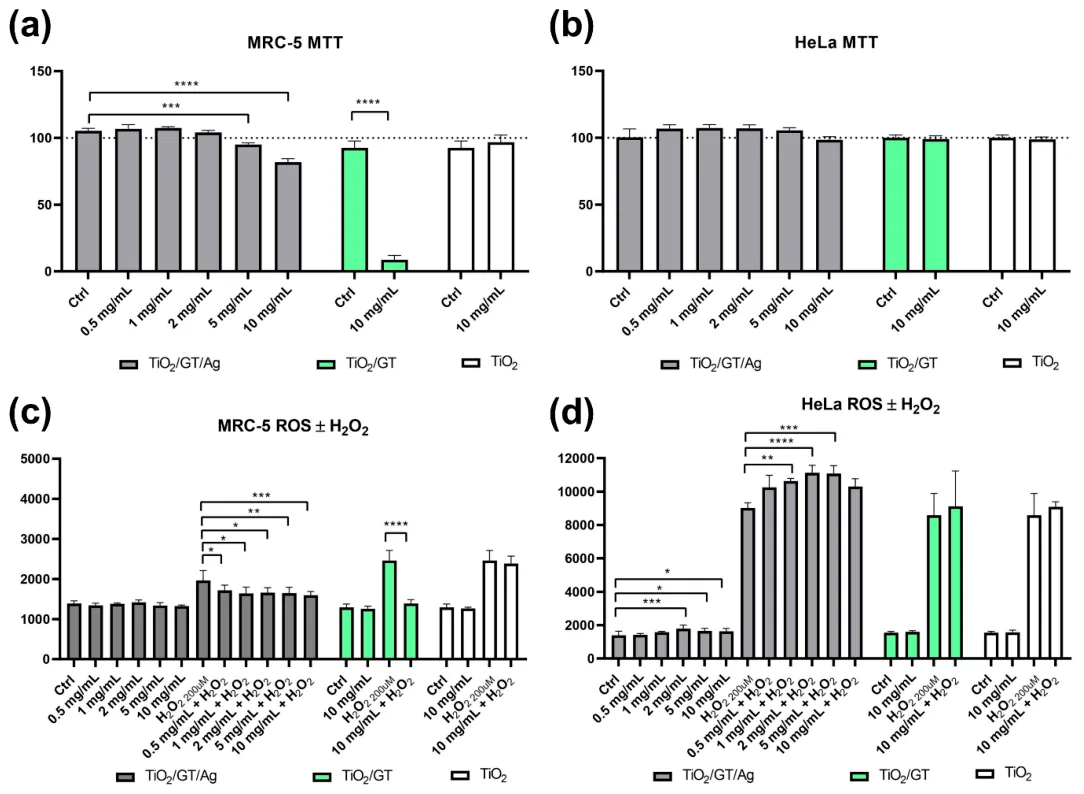
Cytotoxicity and intracellular ROS production induced by TiO_2_, TiO_2_/GT, and TiO_2_/GT/Ag in MRC-5 and HeLa cells after 24 h of exposure. (**a**,**b**) Cell viability assessed by MTT assay in MRC-5 and HeLa cells. (**c**,**d**) Intracellular ROS levels measured using the H_2_DCFDA assay in the absence and presence of H_2_O_2_. Data are presented as mean ± SD (*n* = 9). Statistical significance is indicated as * *p* < 0.05, ** *p* < 0.01, *** *p* < 0.001, **** *p* < 0.0001.

The cytotoxic effects of TiO_2_, TiO_2_/GT, and TiO_2_/GT/Ag were evaluated in MRC-5 and HeLa cells using the MTT assay after 24 h of exposure (Figure 10a,b). Cell viability is expressed as a percentage relative to untreated controls. Treatment with TiO_2_/GT/Ag induced modest, concentration-dependent cytotoxicity in MRC-5 cells, with a 5% reduction in viability at 5 mg/mL and an 18% reduction at 10 mg/mL, whereas HeLa cells showed no significant effects across the tested concentration range. In contrast, exposure of MRC-5 cells to components released from TiO_2_/GT at 10 mg/mL resulted in pronounced cytotoxicity, reducing cell viability by approximately 92%. Notably, HeLa cells treated with TiO_2_/GT under the same conditions showed no reduction in viability. Pristine TiO_2_ did not induce cytotoxic effects in either cell line at concentrations up to 10 mg mL^−1^.

ICP analysis confirmed a Ag loading of 5.1 wt% in the TiO_2_/GT/Ag, corresponding to approximately 0.025 mg mL^−1^ of silver at the lowest tested composite concentration (0.5 mg mL^−1^). At this concentration, neither MRC-5 nor HeLa cells showed reduced viability, indicating good cytocompatibility of the Ag-containing nanocomposite under the investigated conditions.

The pronounced cytotoxicity of the TiO_2_/GT toward MRC-5 cells, together with the substantially lower cytotoxicity of TiO_2_/GT/Ag, suggests that the biological response is determined by the combined physicochemical properties of the released components rather than by the presence of silver alone. Although silver species may exert cytotoxic effects depending on their concentration, chemical form, and bioavailability, incorporating Ag into the TiO_2_/GT composite may modify the material’s surface properties and release profile, potentially affecting the availability of cytotoxic green tea-derived components. In particular, the high cytotoxicity observed for TiO_2_/GT in MRC-5 cells may be associated with the concentration of bioactive green tea-derived compounds released from the material. Previous studies have shown that the biological effects of green tea polyphenols, particularly epigallocatechin-3-gallate (EGCG), strongly depend on concentration and cellular context. For example, high concentrations of EGCG have been shown to markedly reduce fibroblast viability, whereas other studies have reported predominantly non-cytotoxic effects depending on the fibroblast source and experimental conditions [40,41]. Therefore, the substantially lower cytotoxicity of TiO_2_/GT/Ag does not necessarily indicate that silver is intrinsically protective, but may instead reflect changes in the overall composition, release, or bioavailability of cytotoxic components following Ag incorporation.

Further, the markedly different responses of MRC-5 and HeLa cells to the TiO_2_/GT indicate a cell-type-dependent susceptibility. While MRC-5 cells exhibited pronounced loss of viability, HeLa cells remained unaffected under the same exposure conditions. These differences may reflect distinct redox homeostasis, metabolic characteristics, stress-response mechanisms, and sensitivity to green tea-derived compounds between normal and cancer cells. Previous studies have shown that fibroblasts can be highly sensitive to elevated concentrations of EGCG, whereas EGCG’s effects on cancer cells may involve different cellular and redox-regulatory mechanisms. Moreover, green tea polyphenols have been reported to differentially regulate antioxidant and stress-response pathways in normal and cancer cells, potentially contributing to their distinct responses to oxidative and metabolic stress [42,43]. Thus, the observed difference between MRC-5 and HeLa cells may reflect their distinct cellular susceptibility to the released GT-derived components rather than a nonspecific cytotoxic effect of the material. Nevertheless, these experiments do not allow us to establish the individual contribution of EGCG or other GT-derived compounds, Ag species, or cell-specific molecular pathways. To determine the mechanisms underlying these differential responses, we would need to quantify the individual components released from the materials and analyze cellular uptake, mitochondrial function, antioxidant signaling, and cell-death pathways.

Intracellular ROS generation was assessed using the H_2_DCFDA assay (Figure 10c,d). In the absence of H_2_O_2_, none of the tested materials significantly affected ROS levels in MRC-5 cells. Similarly, TiO_2_ and TiO_2_/GT did not alter ROS production in HeLa cells, whereas TiO_2_/GT/Ag induced a slight but significant concentration-dependent increase, indicating a pro-oxidative effect of the Ag-containing composite in cancer cells at concentrations of 2–10 mg mL^−1^. Although intracellular ROS levels were elevated in HeLa cells following treatment with the Ag-containing composite, no statistically significant changes in cell viability were detected by the MTT assay. These findings suggest that the increased ROS generation was not accompanied by a measurable loss of metabolic activity under the experimental conditions employed. The increased intracellular ROS levels observed in HeLa cells, without a corresponding reduction in cell viability, suggest that oxidative stress is an early cellular response that precedes detectable metabolic impairment under the present experimental conditions. Similar temporal discrepancies between ROS generation and cytotoxicity have been reported for silver-containing nanomaterials [44]. Longer exposure times and additional mechanistic studies are needed to further clarify this relationship.

Under H_2_O_2_-induced oxidative stress, distinct cell-type-dependent responses were observed. In MRC-5 cells, TiO_2_ did not alter ROS levels, whereas TiO_2_/GT significantly decreased H_2_O_2_-induced ROS, indicating strong antioxidative activity; however, this concentration also markedly decreased cell viability, which may likewise be linked to the observed ROS reduction. This suggests that mechanisms other than oxidative stress may also contribute to the observed cytotoxic response. TiO_2_/GT/Ag also reduced ROS levels in MRC-5 cells in a concentration-dependent manner, with the strongest effect observed at 10 mg mL^−1^. In contrast, in HeLa cells, co-treatment with TiO_2_ or TiO_2_/GT did not significantly alter H_2_O_2_-induced ROS production, whereas TiO_2_/GT/Ag further enhanced oxidative stress, peaking at 5 mg mL^−1^. The slight decrease at 10 mg mL^−1^ likely reflects cell damage from excessive ROS accumulation.

The differential response of MRC-5 and HeLa cells may reflect differences in their intrinsic redox homeostasis and antioxidant-response mechanisms. Previous studies have shown that green tea polyphenols, particularly EGCG, can differentially regulate antioxidant and stress-response pathways in normal and cancer cells. EGCG has been reported to positively regulate the p62–KEAP1–NRF2–HO-1 axis in normal cells, whereas this pathway may be negatively regulated in cancer cells, contributing to selective biological effects [42]. Similarly, fibroblasts have been shown to exhibit greater resistance to EGCG-induced oxidative stress, accompanied by induction of antioxidant-defense genes, whereas cancer cells displayed mitochondrial ROS generation and downregulation of several antioxidant-related genes [43]. Thus, the reduction in ROS observed in MRC-5 cells following TiO_2_/GT/Ag treatment may reflect the combined contribution of the radical-scavenging properties of the green tea-derived surface components and the more effective antioxidant response of non-malignant cells. Conversely, in HeLa cells, Ag incorporation may promote additional ROS generation and overwhelm cellular antioxidant defenses, resulting in a net pro-oxidant response. This interpretation is consistent with the EPR findings, which demonstrated radical-scavenging activity associated with the green tea-derived surface ligands, while the presence of Ag partially restored oxidative activity. Nevertheless, because the present study did not directly assess antioxidant signaling pathways and mitochondrial ROS, these mechanisms remain hypothetical and warrant further investigation.

## 3. Materials and Methods

### 3.1. Material

Silver(I) nitrate (AgNO_3_, highest available purity grade; Alfa Aesar, Heysham, Lancashire, UK) was used as received without further purification. Titanium dioxide nanoparticles (TiO_2_ NPs; Aeroxide P25, Acros Organics, Geel, Belgium) were used as the photocatalyst material. Milli-Q deionized water (resistivity 18.2 MΩ cm) was used throughout all experimental procedures. For HPLC analysis, HPLC-grade water and methanol (Thermo Fisher Scientific, Waltham, MA, USA) were used as mobile phase components. Reference standards included (−)-epigallocatechin gallate (EGCG, ≥88%) and caffeine (CF > 98.0%) (TCI, Tokyo, Japan).

### 3.2. Preparation of Green Tea Extract

A total of 4 g of dried GT material was placed into 300 mL Erlenmeyer flasks. The material was moistened with 8 mL of distilled water (solid-to-liquid ratio 1:2) and sterilized in an autoclave at 121 °C for 15–20 min after the onset of steam. After sterilization, 92 mL of distilled water was added to each flask, bringing the total volume to 100 mL per 4 g of dry material. Microwave-assisted extraction (MAE) was performed as follows: 3 min at 90 W, followed by 1 min at 180 W. To ensure homogeneous heating, two Erlenmeyer flasks were placed in the microwave oven simultaneously. After cooling to room temperature, the extracts were filtered twice under vacuum, first through a dense filter paper and then through a standard filter paper. For subsequent extractions, vacuum filtration was performed twice using only the standard filter paper. The obtained GT extracts were stored at 4 °C and used within 4 days.

### 3.3. Synthesis of TiO_2_/GT and TiO_2_/GT/Ag Nanocomposite

**Scheme 2:**
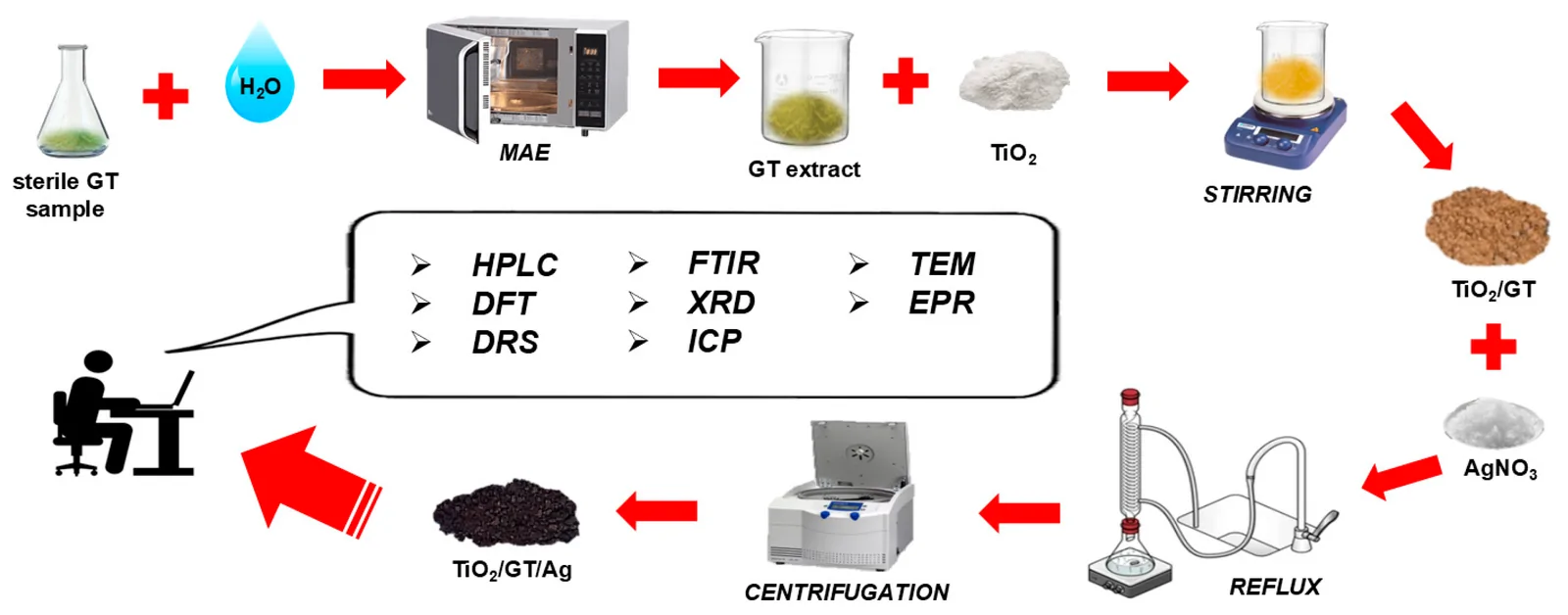
Schematic representation of the experimental procedure used for TiO_2_ functionalization with green tea extract and subsequent silver incorporation.

Scheme 2 schematically illustrates the overall experimental procedure, including TiO_2_ functionalization with green tea extract and subsequent silver incorporation. For the synthesis of the TiO_2_/GT nanocomposite, 200 mL of the prepared GT extract was transferred into a beaker, followed by the addition of 400 mg of TiO_2_ nanoparticles. The suspension was magnetically stirred for 3 h at room temperature and covered with parafilm to prevent airborne contamination. After stirring, the beaker was kept in the dark and left to stand for 3 days. Subsequently, the suspension was centrifuged and washed 4 times with distilled water. The resulting solid was dried in an oven at 40 °C and ground into a fine powder for further experimentation.

Surface functionalization of the TiO_2_/GT nanocomposite with silver was performed as follows. A total of 200 mg of TiO_2_/GT was dispersed in 50 mL of deionized water and transferred into a 100 mL round-bottom flask. The suspension was heated to 60 °C under reflux in an oil bath with continuous magnetic stirring. Subsequently, 500 µL of 0.1 M NaOH and 20 mg of AgNO_3_ were added to the reaction mixture. Upon the addition of AgNO_3_, a rapid darkening of the suspension was observed, indicating the formation of silver-containing domains on the nanocomposite surface. The reaction mixture was refluxed at 60 °C overnight. After cooling to room temperature, the product was centrifuged and washed 4 times with distilled water. Finally, the obtained solid was dried in an oven at 40 °C and ground into a fine powder for further experimentation.

### 3.4. Characterization of TiO_2_/GT and TiO_2_/GT/Ag Nanocomposite

The structural and chemical features of the synthesized nanocomposites were characterized using attenuated total reflectance Fourier transform infrared (ATR-FTIR) spectroscopy over the range 4000–500 cm^−1^ with a spectral resolution of 2 cm^−1^ on a Thermo Scientific Nicolet 6700 FT-IR spectrometer (Thermo Scientific, Waltham, MA, USA). Morphological and microstructural analyses were performed using high-resolution transmission electron microscopy (HRTEM) on a Talos F200X microscope (FEI, Brno, Czech Republic) operated at 200 kV. The instrument is equipped with a Super-X energy-dispersive X-ray spectroscopy (EDS) system comprising four silicon drift detectors (SDDs), enabling elemental analysis and mapping. Diffuse reflectance UV-Vis spectra (DRS) were recorded using a Shimadzu UV-2600 spectrophotometer equipped with an ISR-2600 Plus integrating sphere to investigate the optical properties of the powders. The crystalline structure of the synthesized nanomaterials was examined by X-ray diffraction (XRD) using a Rigaku SmartLab diffractometer with Cu Kα_1,2_ radiation (λ = 0.1540 nm), collected over the 2θ range of 10–90° with a step size of 0.02° and a scan rate of 1° min^−1^.

High-performance liquid chromatography (HPLC) analysis was performed using a Dionex Ultimate 3000 system (Thermo Scientific, Waltham, MA, USA) equipped with a reverse-phase C18 column (ZORBAX Eclipse Plus, 150 mm × 4.6 mm, 5 µm particle size). Gradient elution was carried out using solvent A (H_2_O with 0.1% HCOOH, *v*/*v*) and solvent B (MeOH with 0.1% HCOOH, *v*/*v*) according to the following program: 0–30 min, linear gradient from 10% to 35% B; 30–30.1 min, linear gradient from 35% to 100% B; 30.1–35 min, isocratic at 100% B; 35–35.1 min, linear gradient from 100% to 10% B; and 35.1–45 min, isocratic at 10% B. The flow rate was set to 1.0 mL min^−1^, and the column temperature was maintained at 30 °C. Injection volumes for samples and standards ranged from 5 to 25 µL. Detection was performed using a UV-Vis detector at 280 nm.

The silver content in the TiO_2_/GT/Ag nanocomposite was determined using Inductively Coupled Plasma Optical Emission Spectroscopy (ICP-OES, Thermo Scientific iCAP 7400, Thermo Scientific, Waltham, MA, USA). Before the measurements, the samples were digested in 5 M HNO_3_, and the release of Ag^+^ ions from the TiO_2_/GT/Ag nanocomposite was measured by ICP-OES.

### 3.5. Computational Details

The Gaussian 09 suite of programs has been used for all DFT computations [45]. We used the [Ti_8_O_12_(OH)_8_] cluster as a model system for computational investigations of the electronic and optical properties of the ICT complex formed upon adsorption of isolated GT molecules (EGCG and CF) onto the TiO_2_ surface. This cluster was generated based on the crystal structure of the TiO_2_ Anatase, according to the (101) plane [46]. Using the CAM-B3LYP (Coulomb-attenuating method) functional [47], which accounts for long-range correction, the ground-state geometries of the EGCG/[Ti_8_O_12_(OH)_6_] and CF/[Ti_8_O_14_(OH)_4_] clusters were optimized in a gas phase. The subsequent frequency calculations showed that all optimized geometries have a minimum with no imaginary frequencies. The Pople’s valence double-ζ polarized 6-31G(d,p) basis set was used for all atoms [48,49]. To maintain the anatase structure of TiO_2_, the atoms in the cluster were fixed during optimization, except for two Ti atoms to which the ligands are coordinated, while the atoms of the ligands were free to relax. The formation energies of the corresponding surface complexes were calculated as

E_form_ = E_ICTcomp_. − E_TiO_2__ − E_L_

where E_ICTcomp_., E_TiO_2__, and E_L_ are the energies of corresponding ICT complexes, a free TiO_2_ cluster, and free ligand molecules, respectively. Negative values of E_form_ correspond to the favored process. The CYL view program [50] was used to present the optimized geometry of the clusters, and the GaussSum [51] software produced DOS/PDOS diagrams.

### 3.6. EPR Measurements

Spin trapping and probe reagents, including 5,5-dimethyl-1-pyrroline N-oxide (DMPO), α-(4-pyridyl 1-oxide)-N-tert-butylnitrone (POBN), 2,2′-azino-bis(3-ethylbenzothiazoline-6-sulfonic acid) diammonium salt (ABTS), potassium persulfate (K_2_S_2_O_8_), 2,2-diphenyl-1-picrylhydrazyl (DPPH), 4-hydroxy-2,2,6,6-tetramethylpiperidin-1-oxyl (TEMPOL), and 4-hydroxy-2,2,6,6-tetramethylpiperidone (TMPO) were obtained from Sigma-Aldrich. DMPO was purified by distillation prior to use and stored at −18 °C, while all other reagents were used as received. The ABTS radical cation (ABTS^•+^) was generated in situ by oxidation of ABTS with K_2_S_2_O_8_. All aqueous solutions were prepared using deionized water, and spectroscopic-grade ethanol (CentralChem, Bratislava, Slovakia) was used to dissolve DPPH.

Photocatalyst samples for EPR experiments included pristine TiO_2_, TiO_2_ modified with green tea extract (TiO_2_/GT), and TiO_2_ modified with green tea extract and silver (TiO_2_/GT/Ag). Aqueous suspensions (1.0 g L^−1^) were freshly prepared, stored in the dark, and mixed with the appropriate spin probe prior to analysis. The suspensions were gently aerated under ambient conditions before EPR measurements.

EPR experiments were performed at room temperature (295 K) using a Bruker EMXPlus spectrometer operating at 100 kHz field modulation and equipped with a high-sensitivity resonator and a quartz flat cell (WG-808-Q, Wilamd, Niwot, CO, USA). Samples were irradiated directly in the resonator using either a 365 nm LED source (5 ± 1.0 mW cm^−2^, Bluepoint LED, Honle UV Technology, Gilching, Germany) or a visible-light source (>420 nm, T = 5600 K, illuminance 180 klx). EPR spectra were recorded in situ 2 min after mixing the photocatalysts with the corresponding spin probes. Each experiment was performed at least in duplicate. Relative concentrations of paramagnetic species were estimated from double-integrated EPR signals. Spectral processing was carried out using WinEPR software, while quantitative analysis and spin-Hamiltonian simulations were performed using EasySpin working under Matlab^®^ [52].

Low-temperature EPR measurements were also conducted at 100 K. Photocatalyst powders were placed in quartz EPR tubes, rapidly frozen in liquid nitrogen at 100 K, and measured in the dark and under irradiation. A Bruker variable-temperature unit controlled the temperature.

### 3.7. Antimicrobial Activity of TiO_2_/GT and TiO_2_/GT/Ag Nanocomposite

The antimicrobial activity of the TiO_2_/GT and TiO_2_/GT/Ag nanocomposites was assessed against three representative pathogenic microorganisms: *Escherichia coli* (Gram-negative bacterium), *Staphylococcus aureus* (Gram-positive bacterium), and *Candida albicans* (yeast). Microbial strains were cultured overnight at 37 °C in tryptone soy broth to obtain a metabolically active inoculum. For antimicrobial testing, defined amounts of nanocomposite powders (5, 10, 25, and 50 mg) were dispersed in 5 mL of sterile physiological saline (0.85% NaCl) and inoculated with approximately 10^5^ CFU mL^−1^ of the respective microorganism. The resulting suspensions were incubated at 37 °C in the dark. At predetermined time points (1, 3, 6, and 24 h), 100 µL aliquots were withdrawn, spread onto tryptone soy agar plates, and incubated for an additional 24 h at 37 °C. After incubation, viable colonies were counted to quantitatively evaluate the nanocomposites’ antimicrobial activity.

### 3.8. Cytotoxicity and ROS Assays of TiO_2_/GT and TiO_2_/GT/Ag Nanocomposite

Human fetal lung fibroblasts (MRC-5, ATCC^®^ CCL-17^TM^, Rockville, MD, USA) and human cervical carcinoma cells (HeLa, ATCC^®^ CCL-2^TM^, Rockville, MD, USA) were cultured in 25 cm^2^ tissue culture flasks under standard conditions (37 °C, 5% CO_2_) using complete RPMI 1640 medium (Biowest, Nuaillé, France) supplemented with 10% fetal calf serum (FCS, Sigma-Aldrich, St. Louis, MO, USA) and 1% antibiotic-antimycotic solution (Sigma-Aldrich, St. Louis, MO, USA). Upon reaching approximately 70% confluence, cells were detached using 0.25% trypsin-EDTA solution (Biowest, Nuaillé, France) and seeded into 96-well plates at a density of 1.5 × 10^4^ cells per well. Cells were allowed to adhere for 24 h prior to treatment.

Stock suspensions of TiO_2_, TiO_2_/GT, and TiO_2_/GT/Ag were prepared by dispersing the dry nanomaterials in complete RPMI 1640 medium at a concentration of 40 mg mL^−1^. The suspensions were incubated for 24 h at 37 °C in a humidified atmosphere with 5% CO_2_ to allow the release of soluble components from the nanoparticles. After incubation, the samples were centrifuged at 300× *g* for 10 min, and the supernatants were collected and diluted with fresh cell culture medium to obtain final concentrations of 0.5, 1, 2, 5, and 10 mg mL^−1^ for the treatments.

For cytotoxicity evaluation, the culture medium was replaced with fresh medium containing the respective nanomaterial-derived supernatants, and cells were incubated for 24 h. After 24 h of incubation, the treatments were removed, cells were washed twice with phosphate-buffered saline (PBS), and 100 µL of fresh medium was added per well. Cell viability was assessed using the MTT assay. Briefly, MTT reagent (thiazolyl blue tetrazolium bromide, 1 mg mL^−1^) was added to each well (10 µL per well) and incubated for 2 h at 37 °C in the dark. The resulting formazan crystals were dissolved in 10% sodium dodecyl sulfate (SDS) in 0.01 M HCl, and absorbance was measured at 570 nm using a microplate reader. Three independent experiments were performed in triplicate (*n* = 9).

Intracellular reactive oxygen species (ROS) production was evaluated using the 2′,7′-dichlorodihydrofluorescein diacetate (H_2_DCFDA) assay. Cells were treated with nanomaterial-derived supernatants for 24 h, after which the treatments were removed, and the cells were washed with PBS. Cells were then incubated with 5 µM H_2_DCFDA in PBS for 45 min in the dark. Following PBS wash, cells were exposed to PBS alone (negative control) or 200 µM H_2_O_2_ (positive control) and incubated for an additional 1 h. The conversion of non-fluorescent H_2_DCFDA to fluorescent 2′,7′-dichlorofluorescein (DCF) was quantified by measuring fluorescence at excitation and emission wavelengths of 485 and 535 nm, respectively. ROS levels were expressed as relative fluorescence intensity. All experiments were performed in triplicate (*n* = 9).

## 4. Conclusions

The present study demonstrates that surface modification of TiO_2_ nanoparticles with green tea-derived surface ligands, followed by silver impregnation, yields a redox-active nanoplatform with distinct light-dependent biological effects. HPLC, FTIR, DRS, and DFT analyses provided consistent evidence of an organic–inorganic interface between TiO_2_ and green tea-derived ligands. DFT calculations further revealed their complementary interfacial roles, with EGCG inducing interfacial charge transfer and caffeine forming the most stable surface complexes. EPR investigations revealed that green tea functionalization suppresses excessive radical formation and provides pronounced radical-scavenging capacity, whereas silver incorporation shifts the redox balance toward oxidative pathways, particularly under irradiation. These fundamental redox differences directly translate into biological performance. The Ag-containing nanocomposite exhibited the strongest overall antimicrobial activity. Visible-light irradiation provided an additional enhancement, primarily against *S. aureus*, suggesting a strain-dependent contribution of photogenerated ROS that act in combination with silver species. In cell-based assays, TiO_2_/GT/Ag exhibited antioxidant activity and reduced H_2_O_2_-induced oxidative stress in non-malignant cells, whereas the Ag composite selectively exacerbated oxidative stress in cancer cells. Altogether, these findings demonstrate that engineering the organic–inorganic interface through green tea-derived ligands and silver incorporation provides an effective strategy for tuning the redox properties and light-responsive biological performance of TiO_2_-based nanomaterials, thereby expanding their potential for antimicrobial and biomedical applications.

## Data Availability

The data that support the findings of this study are available from the corresponding author upon reasonable request.

## Supplementary Materials

The following supporting information can be downloaded at https://www.mdpi.com/article/10.3390/molecules31173123/s1. Figure S1. (a) The optimized geometry and (b) Total density of states (TDOS) of EGCG(B)/[Ti_8_O_12_(OH)_6_] complex. (gray–carbon; white–hydrogen; red–oxygen; large gray–titanium). Figure S2. Experimental EPR spectrum of GT measured in water suspension (1 g L^−1^). Figure S3. Time dependence of the EPR intensity of the second line of the EPR spectra of •POBN-OH. The signal increases and then disappears with the LED@365nm. Figure S4. The concentration of TEMPONE monitored during the continuous UV (LED@365nm) exposure as a result of TMPO photooxidation (c0,TMPO = 0.01 M, water) evaluated from double-integrated EPR spectra monitored in the aqueous aerated suspensions of pristine and modified TiO_2_ (GT or GT/Ag) during the continuous LED@365nm irradiation for 180 s (photocatalyst loading 0.4 g L^−1^). Figure S5. Time dependence of DPPH radical relative concentration measured in the dark or under LED@365nm exposure evaluated from double-integrated EPR spectra monitored in the aqueous aerated suspensions of pristine and modified TiO_2_ (GT or GT/Ag). or GT measured in dark or (a) during the continuous LED@365nm irradiation. Figure S6. The comparison of DPPH radical termination (in %) in dark or under UV (a) and VIS (b) exposure.
