# Supplementary material for: Interfacial Redox Engineering of TiO2 Nanocomposites Using Green Tea-Derived Ligands and Silver
Source: Molecules. 2026 Sep 6;31(17):3123. doi: 10.3390/molecules31173123 (PMC13567241; doi:10.3390/molecules31173123)
Supplement: Supplementary file 1 [file molecules-31-03123-s001.zip › molecules-4504755-supplementary.pdf]

molecules

Supporting Information

Interfacial Redox Engineering of TiO_2 Nanocomposites Using Green Tea-Derived Ligands and Silver

Valentina Nikšić [1], Dušan Sredojević [1], Miriama Malček Šimunková [2], Andrea Pirković [3], Ana Milivojević [4], Vlasta Brezová [2] and Vesna Lazić [1,*]

1 Centre of Excellence for Photoconversion, Vinča Institute of Nuclear Sciences, University of Belgrade, 11000 Belgrade, Serbia; valentina.niksic@vin.bg.ac.rs (V.N.)
2 Department of Physical Chemistry, Institute of Physical Chemistry and Chemical Physics, Faculty of Chemical and Food Technology, Slovak University of Technology in Bratislava, 812 37 Bratislava, Slovakia; miriama.simunkova@stuba.sk (M.M.Š.); vlasta.brezova@stuba.sk (V.B.)
3 INEP Institute for Application of Nuclear Energy, University of Belgrade, 11000 Belgrade, Serbia; andrea.pirkovic@inep.co.rs
4 Faculty of Technology and Metallurgy, University of Belgrade, 11000 Belgrade, Serbia
* Correspondence: vesna.lazic@vin.bg.ac.rs

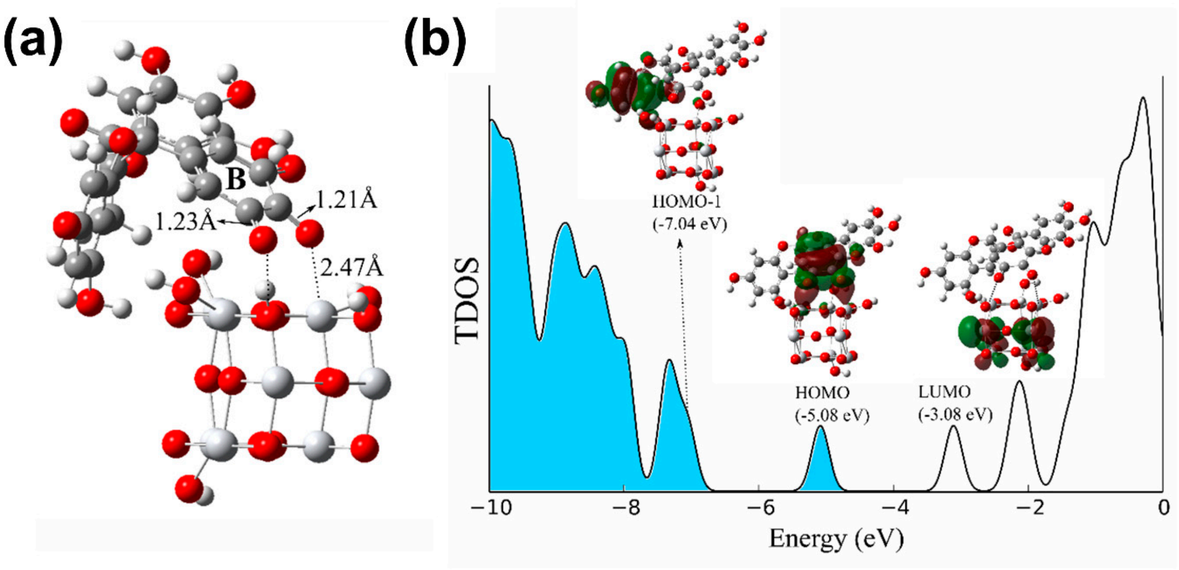

Figure S1. (**a**) The optimized geometry and (**b**) Total density of states (TDOS) of EGCG(B)/[$Ti_8O_{12}(OH)_6$] complex. (gray – carbon; white – hydrogen; red – oxygen; large gray – titanium).

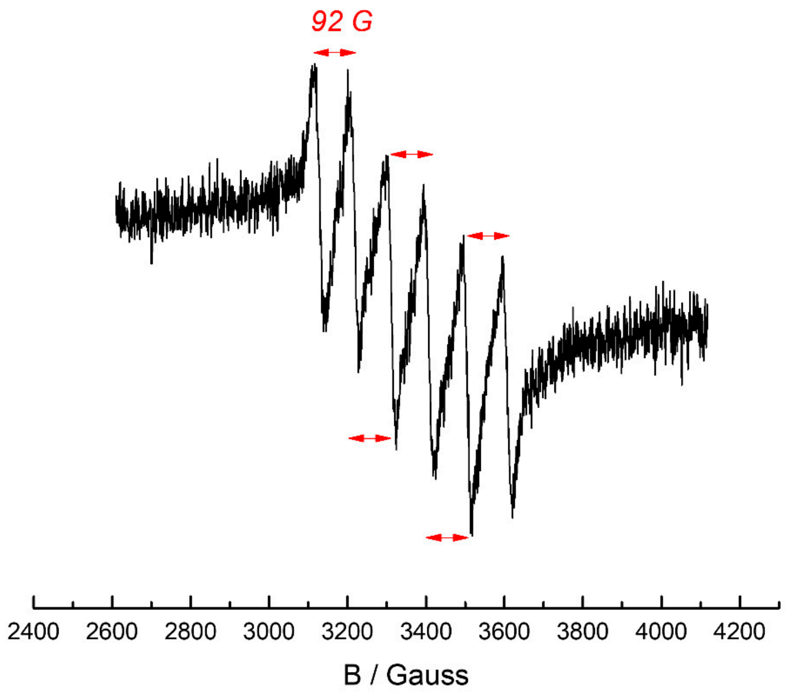

Figure S2. Experimental EPR spectrum of GT measured in water suspension (1 g L^{-1}). SW = 1500 G, hfcc 92 G.

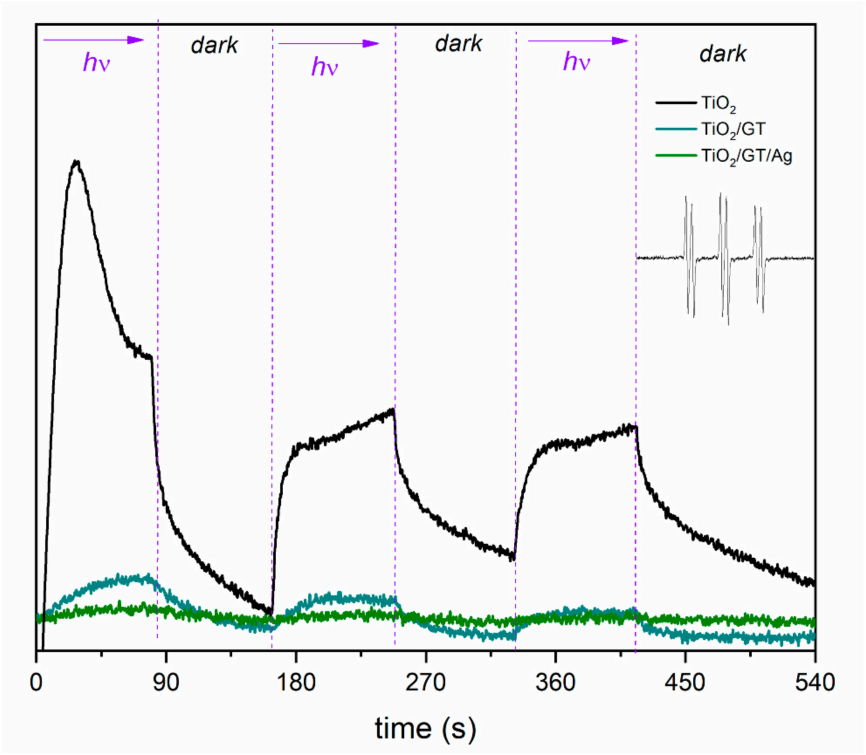

Figure S3. Time dependence of the EPR intensity of the second line of the EPR spectra of •POBN-OH. The signal increases and then disappears with the LED@365nm. Irradiation was turned on and off at the indicated time intervals. c(DMPO); c(POBN) = 5 mM; photocatalyst loading (0.25 g L^{-1}).

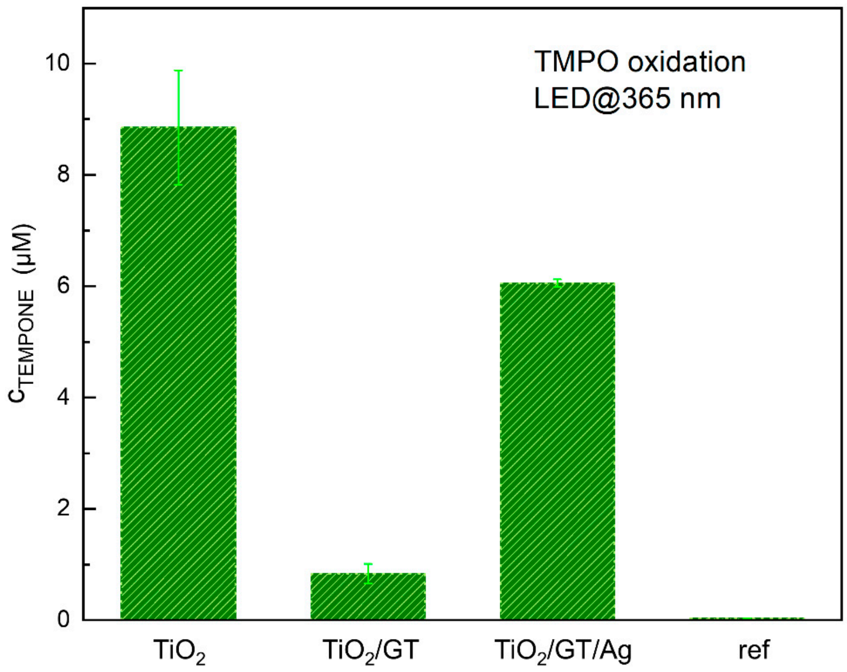

Figure S4. The concentration of TEMPONE monitored during the continuous UV (LED@365nm) exposure as a result of TMPO photooxidation ($c_{0,TMPO}$ = 0.01 M, water) evaluated from double-integrated EPR spectra monitored in the aqueous aerated suspensions of pristine and modified TiO_2 (GT or GT/Ag) during the continuous LED@365nm irradiation for 180s (photocatalyst loading 0.4 g L^{-1}).

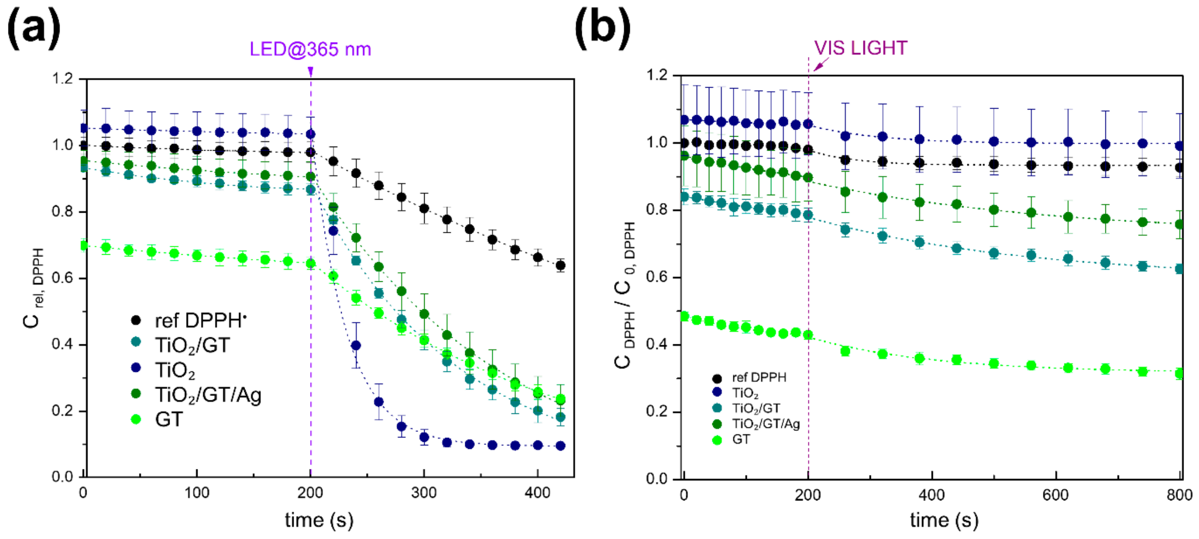

Figure S5. Time dependence of DPPH radical relative concentration measured in the dark or under LED@365nm exposure evaluated from double-integrated EPR spectra monitored in the aqueous aerated suspensions of pristine and modified TiO_2 (GT or GT/Ag). or GT measured in dark or a) during the continuous LED@365nm irradiation ($c_{(DPPH)}$ = 0.09 mM; photocatalyst loading 0.09 g L^{-1}; * extract loading 0.009 g L^{-1}); b) during the continuous VIS light irradiation ($c_{(DPPH)}$ = 0.045 mM; ethanol: water = 9:1, v:v; photocatalyst loading (0.09 g L^{-1}); *extract loading (0.009 g L^{-1}). The symbols represent experimental data, while the dotted lines show their least-squares fits.

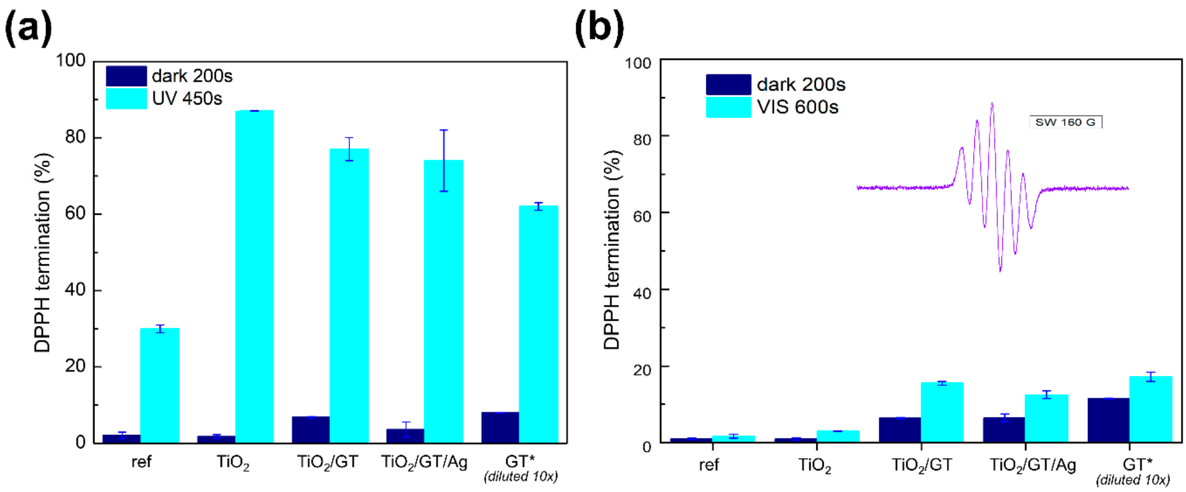

Figure S6. The comparison of DPPH radical termination (in %) in dark or under UV (a) and VIS (b) exposure. Inset in Figure b) EPR spectrum (quintet) monitored for DPPH radical measured ($c_{(DPPH)}$= 45 μM, ethanol: water = 9:1, v:v), magnetic field sweep: 160 G.

Disclaimer/Publisher's Note: The statements, opinions and data contained in all publications are solely those of the individual author(s) and contributor(s) and not of MDPI and/or the editor(s). MDPI and/or the editor(s) disclaim responsibility for any injury to people or property resulting from any ideas, methods, instructions or products referred to in the content.
